# Variation characteristics of stress distribution in the subchondral bone of the knee joint of judo athletes with long-term stress changes

**DOI:** 10.3389/fendo.2022.1082799

**Published:** 2023-01-24

**Authors:** Zhiqiang Li, Guanghua Xu, Chengjun Wang, Qiuyuan Wang, Caiping Liu, Tingting Guo, Lijun Wu, Diankang Cao

**Affiliations:** ^1^ School of Physical Education, North University of China, Taiyuan, China; ^2^ Guangdong Engineering Research Center for Translation of Medical 3D Printing Application, Guangdong Provincial Key Laboratory of Medical Biomechanics, National Key Discipline of Human Anatomy, School of Basic Medical Sciences, Southern Medical University, Guangzhou, China; ^3^ Orthopedic Center, Affiliated Hospital of Guangdong Medical University, Zhanjiang, China; ^4^ Graduate School of Beijing University of Chinese Medicine, Beijing, China; ^5^ Ergonomics and Functional Clothing Laboratory, School of Textiles and Fashion, Shanghai University of Engineering Science, Shanghai, China; ^6^ School of Physical Education, Shanxi University, Taiyuan, China

**Keywords:** Judo, distal femur, tibia plateau, stress, high density area, judo technique, computed tomography osteoabsorptiometry

## Abstract

**Objective:**

To investigate the distribution of bone density in the subchondral bone tissue of the knee joint due to the mechanical stress load generated by judo, the bone tissue volume of different densities and the bone remodeling characteristics of the subchondral bone of the knee joint.

**Methods:**

CT imaging data of the knee joint were collected from 15 healthy individuals as controls and 15 elite judo athletes. Firstly, they were processed by the CTOAM technique, and secondly, the distribution pattern of high-density areas of the knee joint was localized using nine anatomical regions. In addition, three tomographic images were selected in the sagittal, coronal, and axial 2D image windows to observe the distribution of different densities of bone tissue. Finally, the percentage of bone tissue volume (%BTV) and bone remodeling trend of bone tissues with different densities were determined.

**Results:**

In this study, high-density areas were found in the 4th, 5th, and 6th regions of the articular surface of the distal femur and the 1st, 2nd, 3rd, 4th, 5th, 6th, 7th and 8th regions of the tibial plateau in judo athletes; the distribution of high-density areas on the articular surface of the distal femur in control subjects was similar with judo athletes, and high-density areas were mainly found in the 4th and 5th regions of the tibial plateau. The %BTV of low (401-500HU in the distal femur; 301-400 HU and 401-500HU in the tibial plateau), moderate, and high bone density was higher in judo athletes than in controls in the subchondral bone of the distal femur and tibial plateau (P< 0.05).

**Conclusion:**

The history of compressive stresses, struck stresses, soft tissue tension and pull, self-gravity and intra-articular stress loading generated by the lower limb exercise technique of judo leads to specific forms of stress distribution and bone tissue remodeling in the subchondral bone tissue within the distal femur and tibia plateau.

## 1 Introduction

Judo is an Olympic sport that originated in Japanese Budo, and its technical elements include standing throwing techniques (*Nage-waza*), ground grappling techniques (*Katame-waza*) and striking techniques (*Atemi-waza*; Banned in judo competition). Judo, one of the world’s most popular competitive martial arts sports, was officially entered into the Olympic Games for both men and women in 1992. Current statistics from the International Judo Federation put the number of participants worldwide at about 40-50 million (1).

According to the injury statistics and epidemiological studies on the official website of the Olympic Games, it was found that judo has intense confrontation and athletes often have a high level of sports injuries during competition, and the location and degree of injuries are related to the athletes’ technical and tactical expertise, seniority, and other factors (2). For example, non-elite athletes are prone to sprains, bruises, and contusions of the limbs, while elite-level athletes more often get sprains and dislocations of sports injuries (2). In addition, the knee, shoulder (3), and finger joints (4, 5) are the most vulnerable areas for judo athletes. Rare and serious injuries such as concussion, spine (cervical; thoracic; or lumbar) injuries, and even death can occur during training competitions. Judo is therefore a competitive sport that is not completely safe. Due to the large number of throws, grappling and joint lock techniques, many knee joint injury cases occur among judo athletes. Therefore, the key mechanism of knee joint injury needs to be further investigated.

Long-term environmental stresses and subchondral bone stresses in joints can be reflected by bone density distribution patterns, which cannot be obtained indirectly using *in vivo* measurements of intra-articular stress distribution without being aggressive to the living subject. An earlier study found that the Computed tomography osteoabsorptiometry (CTOAM) technique could directly obtain the internal joint stress distribution patterns of subjects and assess the characteristics of changes in intra-articular stress distribution after surgery or injury (6). Therefore, the use of CTOAM technique to detect subchondral bone density (stress) distribution in the knee joint of judo athletes is relatively more acceptable. Currently, however, there are insufficient imaging studies in the field of sports medicine regarding the distribution and changes of lower limb bone morphology and bone tissue density in judo athletes. Therefore, the purpose of this study was using the CTOAM technique combined with the characteristics of judo techniques to analyze the pattern of bone tissue distribution, volume percentage changes, and characteristics of high-density femoral remodeling areas within the subchondral bone of the distal femur and tibia plateau in two groups of subjects. We hypothesized that ground reaction forces, gravity, impact forces, intra-articular and joint muscle movement stresses generated by judo techniques may be responsible for the specific stress distribution and bone tissue-specific remodeling in the knee joints of judo athletes.

## 2 Methods

### 2.1 Data collection

This study was approved for implementation by the authors’ institution. The consents of all subjects were obtained and informed consent forms were signed. We measured age, height, weight, and foot length in 15 elite judo athletes and 15 normal adult males (**Table 1**). All athletes had a training history of 6 years or more, with right dominance. The normal subjects had no history of sports participation or special training. All subjects had no history of injury, discomfort or pain in the knee joint. As a part of the experiment, CT scans of lower limbs were carried out for all subjects and informed consent was obtained for subjects (Revolution CT, GE healthcare, Waukesha, WI USA; tube voltage 120kv; layer thickness 0.625mm; tube current automatically adjusted 100-400mA).

**Table 1:** Basic characteristics of judo and control group.

	Judo	Control group	*P* value
Number	15	15	/
Sex	Male	Male	/
Dominant aspect	Right	Right	/
Training age(yrs)	6	/	/
Age(yrs)	20.9 ± 2.5	20.133 ± 1.5	.299
Stature(cm)	177.9 ± 6.2	175.333 ± 4.1	.189
Weight(kg)	78.4 ± 12.6	76.300 ± 3.1	.536
Shoes size (Europe)	42.2 ± 1.5	42.800 ± 0.9	.196

### 2.2 CTOAM

First, we used Materialise Mimics 21.0 (Materialize, Leuven, Belgium) to screen and preprocessed the DICOM format images, and used the high-density (Hounsfield units, HU) regions of the subchondral bone of the distal femur and tibial plateau in the images as regions of interest (ROI). Second, we defined the subject’s subchondral bone density range of the distal femur and tibial plateau as 12 density intervals, and divided each interval into a different color (6, 7). Finally, we superimposed the image data for each density interval to obtain a complete pseudo-color image of the subchondral bone density of the knee joint.

### 2.3 Distribution patterns of bone tissue with different densities within the subchondral bone of the knee joint in the judo and control group

First, we used the mask editing function in Materialise Mimics software to obtain the topographic data of bone density around the epiphyseal line of the knee joint (distal femur and tibial plateau). Next, the topographic image data of bone density around the epiphyseal line of the knee were extracted using the area growth function. In addition, we use the mask erase function in the software to erase layer by layer the non-interesting mask image data outside the knee epiphyseal line. Then we convert the preserved mask (ROI area) into the corresponding bone density model. Among them, the corresponding bone models were adjusted to highly transparent, moderately transparent, and non-transparent using the visible function and transparency function in the 3D viewport. For example, the low and moderate bone density bone tissues were used as highly transparent models; the high bone density was used as a moderately transparent model, and the very high bone density tissues (1201HU<) were used as non-transparent models. Finally, we took sectional images of the judo group and the control group to evaluate the subchondral bone mineral density distribution pattern of the knee joint.

### 2.4 Evaluation of the distribution pattern of high bone density areas on the knee joint surface in the judo and control group

We divided the reconstructed bone tissue models of the proximal femur and tibial plateau into nine anatomical regions. The frequency, distribution, and percentage number of high-density zones appearing in the subchondral bone region of the knee were counted.

### 2.5 Quantitative analysis of the volume percentage of the knee subchondral bone in the judo and control group

**Figure 1:**
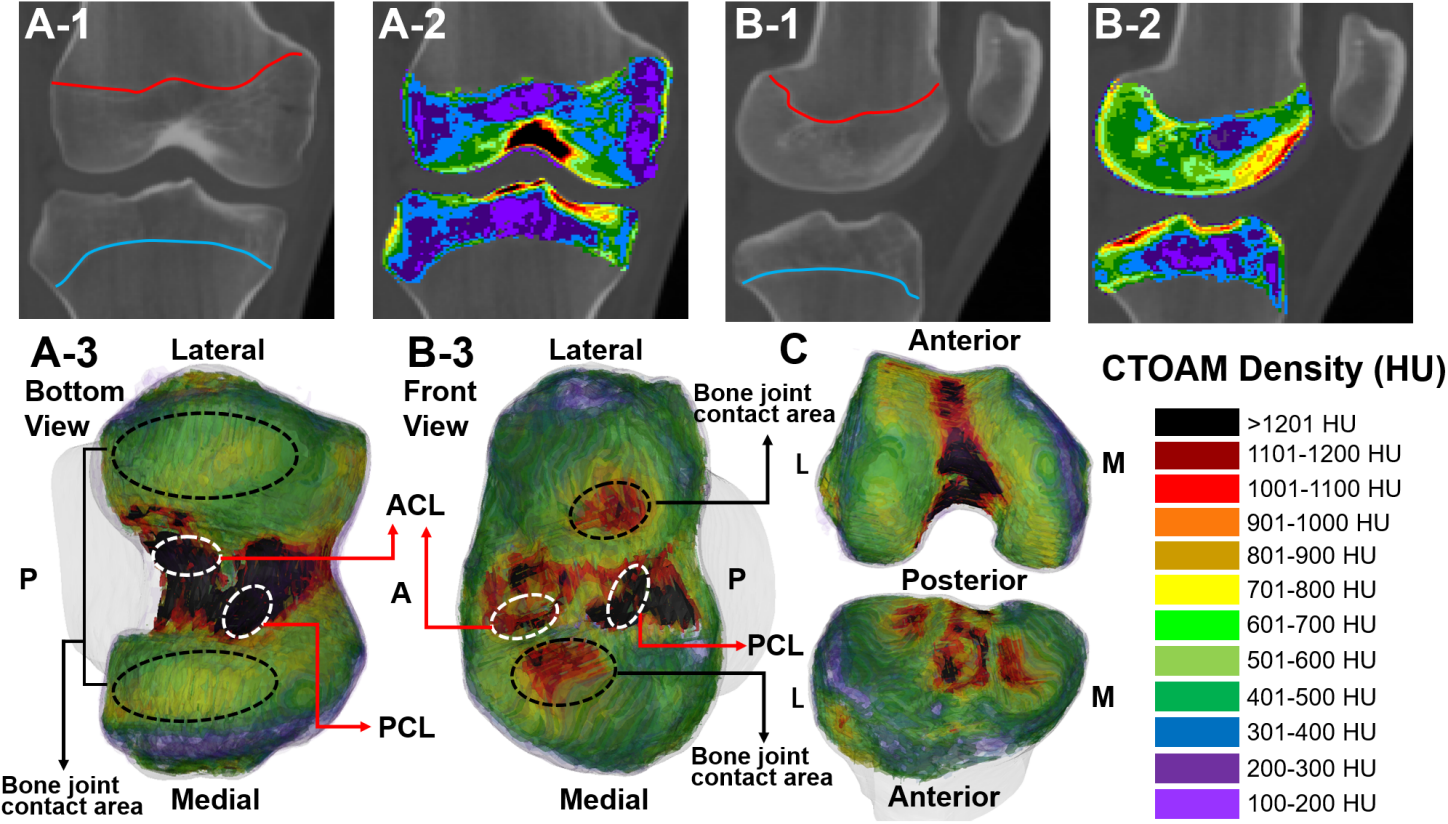
Range and post-processing reconstructed model of subchondral bone image segmentation (CT value, Hounsfield Unit, HU) of the knee joint in two subjects. **(A-1, B-1)** CT segmentation range of the knee the epiphyseal line; **(A-2, B-2)** CTOAM segmentation images of the knee epiphyseal line; **(A-3, B-3)** CTOAM model of the reconstructed bone tissue of the knee joint; **(C)** Reconstructed knee joint model after opening (front view); CTOAM image density interval. These pictures were from the judo athletes. ACL is anterior cruciate ligament; PCL is posterior cruciate ligament; M is medial; L is lateral.


Firstly, we reconstructed the subchondral bone tissues between the articular surfaces of the distal femur and the tibial plateau to the epiphyseal line as 3D models, respectively, and obtained the corresponding bone tissue model volumes one by one. Secondly, we summed the volumes of different densities of bone tissues within their articular surfaces, and finally obtained the total volume of subchondral bone tissue within the distal femur and tibial plateau. Finally, the %BTV within the knee joint surface was defined as %BTVi with i being 1 of the 12 density rangers. The %BTVi was obtained by dividing the volume of a certain density of bone tissue within the distal femur and tibial plateau by the corresponding total volume of bone tissue. We used the subchondral bone tissue (the epiphyseal line to articular surface) within the knee joint surface in both groups of subjects as the bone tissue of interest (BTOI), and then we analyzed the distribution and Volumetric percentage trends of different densities of subchondral bone tissue within the knee joint surface. This was achieved by comparing the differences in the %BTVi of subchondral bone tissue (7) (**Figure 1**). Generally, CT image segmentation is used to obtain complete human organs or tissue images (organ density ranges from the lowest to the highest), such as complete femur, tibia, pelvis or spine. CTOAM technology divides the CT value of bone tissue into different density intervals, and applies different colors to bone tissue in different density intervals to indicate the distribution of bone density.

$$\%BTVi=\frac{volume\;of\;bone\;tissue\;with\;a\;certain\;density\;in\;distal\;femur\;or\;tibial\;plateau}{total\;volume\;of\;subchondral\;bone\;tissue\;of\;the\;distal\;femur\;or\;tibial\;plateau}$$

### 2.6 Statistical analysis

To determine whether the data from the judo and control group conformed to a normal distribution, Kolmogorov-Smirnov was first performed. For comparison of judo and control groups, the Student t-test was used if the data fit a normal distribution. A nonparametric test was used to compare groups and within groups in cases where the distribution was not normal. statistical results were expressed as mean ± SD, with P< 0.05 being statistically significant. IBM SPSS Statistics 25.0 software was used.

## 3 Results

### 3.1 Comparison of basic characteristics between groups

There were no statistical differences in age, height, weight, and foot size in the elite group of judo athletes compared to the control group, and data on subject characteristics are presented in **Table 1**.

### 3.2 Evaluation of the distribution pattern of different densities of bone tissue under the knee articular cartilage in tomographic imaging

**Figure 2:**
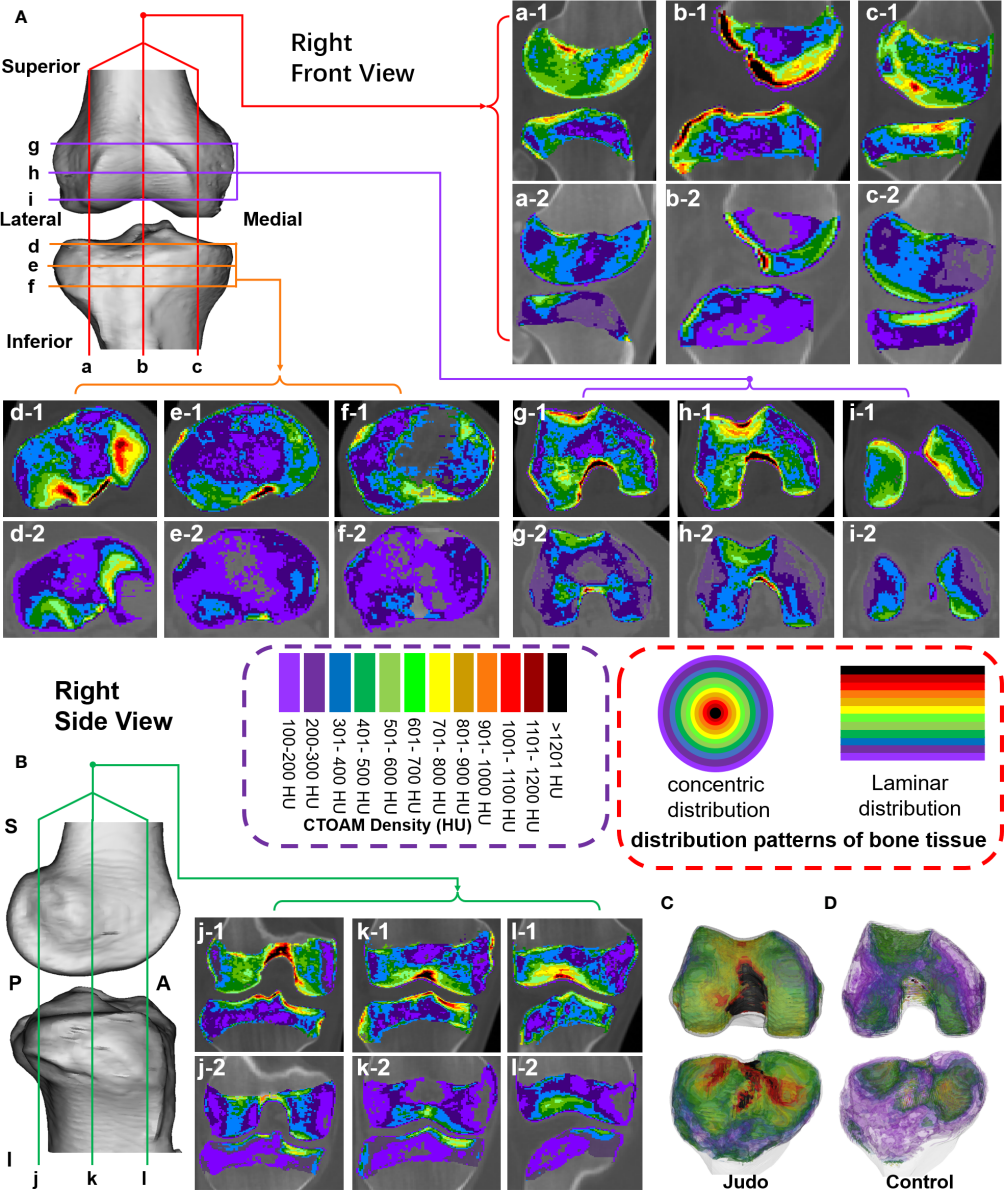
Tomographic images of the distribution of bone tissue with different bone density in the subchondral bone of the knee joint surface in two groups of subjects. **(A)** Sagittal and axial tomographic sections of the knee joint; **(B)** Coronal sections of the knee joint; **(C, D)** Superimposed images of different bone density in the knee joint of judo athletes and control subjects. **(1-a–c-1)** are sagittal images of judo athletes; **(d-1–f-1)** are axial images of tibial plateau of judo athletes; **(g-1–i-1)** are axial images of distal femur of judo athletes; **(j-1–l-1)** are coronal images of judo athletes. **(a-2–c-2)** are sagittal images of control subjects; **(d-2–f-2)** are axial images of tibial plateau of control subjects; **(g-2–i-2)** are axial images of distal femur of control subjects; **(j-2–l-2)** are coronal images of control subjects.

In the axial, sagittal and coronal section images, the subchondral bone sections of the knee joints of judo athletes showed concentric distribution. The ligament initiation and termination areas, osteoarticular contact areas and the epiphyseal line areas had higher densities; whereas the control group showed a laminar distribution with lower density in the osteoarticular contact areas and even lower density in the non-direct contact areas (**Figure 2**).

### 3.3 Comparison of frequency and distribution patterns of the high-density zone of the knee articular surface

**Figure 3:**
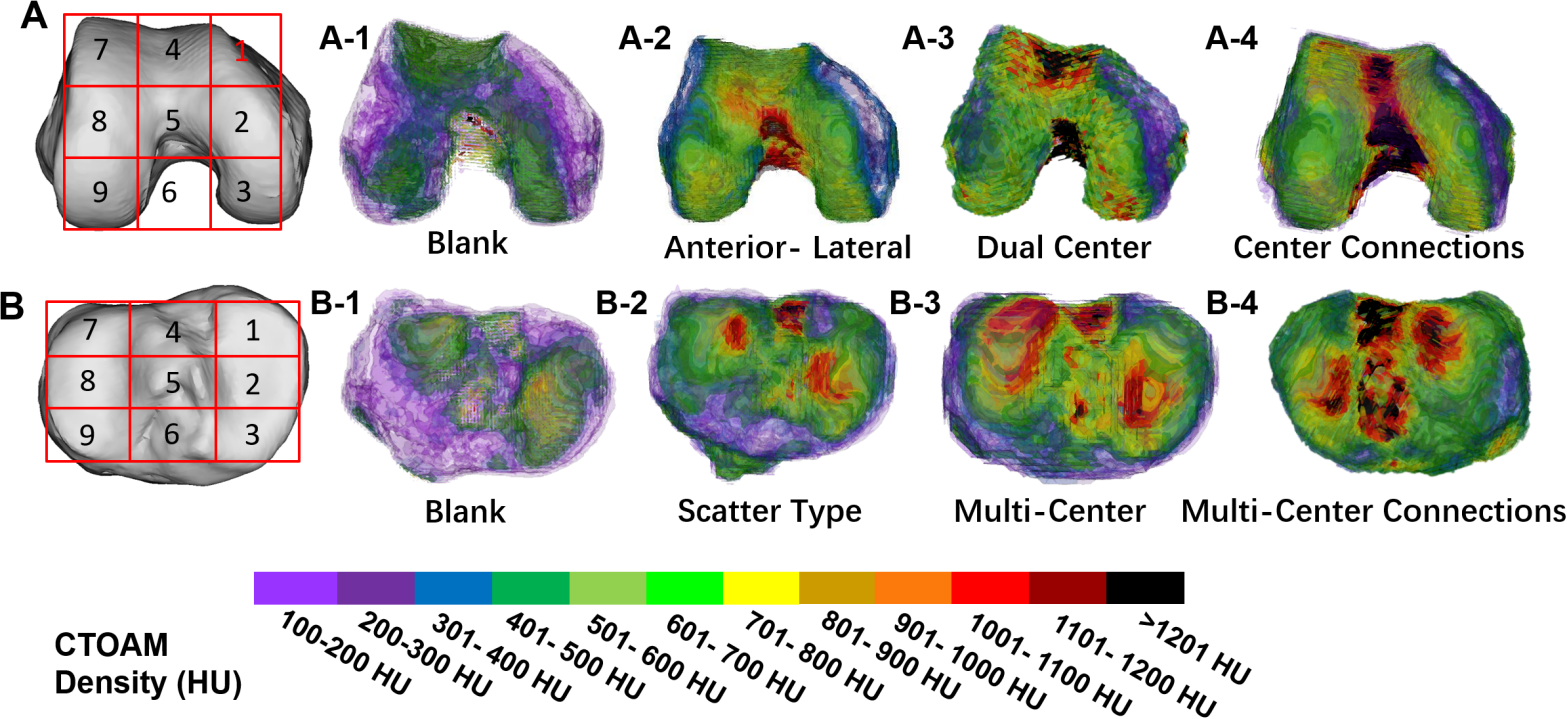
**(A)** is the anatomical division pattern of the high-density area of the distal femur; **(B)** is the anatomical division pattern of the high-density area of the tibial plateau. bone density distribution pattern in the knee joint in two subject groups. **(A1–A-4)** provide an overview of the distribution types of high-density areas on the articular surface of the distal femur; **(B1–B4)** provide an overview of the high-density zones distribution types on the articular surface of the tibial plateau.

The measurement patterns of the high-density areas of the knee joints in both groups are shown in **Figure 3**. The results of the analysis of the location and distribution patterns of the high-density areas of the proximal tibia and distal femur in both groups are detailed in **Table 2**. In the distribution patterns of the distal femur, the subjects in both groups showed blank type, anterior-lateral, dual center type, and center connections type. In the distribution pattern of the tibial plateau, the subjects showed blank type, scatter type, multi-centers type, and multi-center connections in **Table 3**.

**Table 2:** knee articular bone density distribution pattern classification data.

Position	Distal Femur				Tibia Plateau			
Groups	JUDO		Control group		JUDO		Control group	
High density area	Number(30)	Frequency(%)	Number(30)	Frequency(%)	Number(30)	Frequency(%)	Number(30)	Frequency(%)
1	0	0%	4	13%	15	50%	7	23%
2	9	30%	2	7%	25	83%	19	63%
3	2	7%	6	20%	18	60%	10	33%
4	24	80%	12	40%	30	100%	20	67%
5	30	100%	30	100%	23	77%	16	53%
6	28	93%	20	67%	24	80%	13	43%
7	3	10%	5	17%	28	93%	15	50%
8	2	7%	0	0%	16	53%	9	30%
9	3	10%	8	27%	0	0%	0	0%

**Table 3:** knee articular distribution pattern of high bone density area classification data.

Position	Distal Femur				Tibia Plateau			
Groups	JUDO		Control group		JUDO		Control group	
High density area	Number(30)	Frequency(%)	Number(30)	Frequency(%)	Number(30)	Frequency(%)	Number(30)	Frequency(%)
Blank	0	0	17	57%	0	0	6	20%
Anterior-Lateral	8	27%	0	0	/	/	/	/
Dual Center	19	63%	9	30%	/	/	/	/
Center Connections	3	10%	4	13%	/	/	/	/
Scatter type	/	/	/	/	17	57%	16	53%
Multi-Center	/	/	/	/	5	17%	3	10%
Multi-Center Connections	/	/	/	/	8	27%	5	17%

### 3.4 Comparison of the volume percentage of the bone density of the knee subchondral bone between the two groups

**Figure 4:**
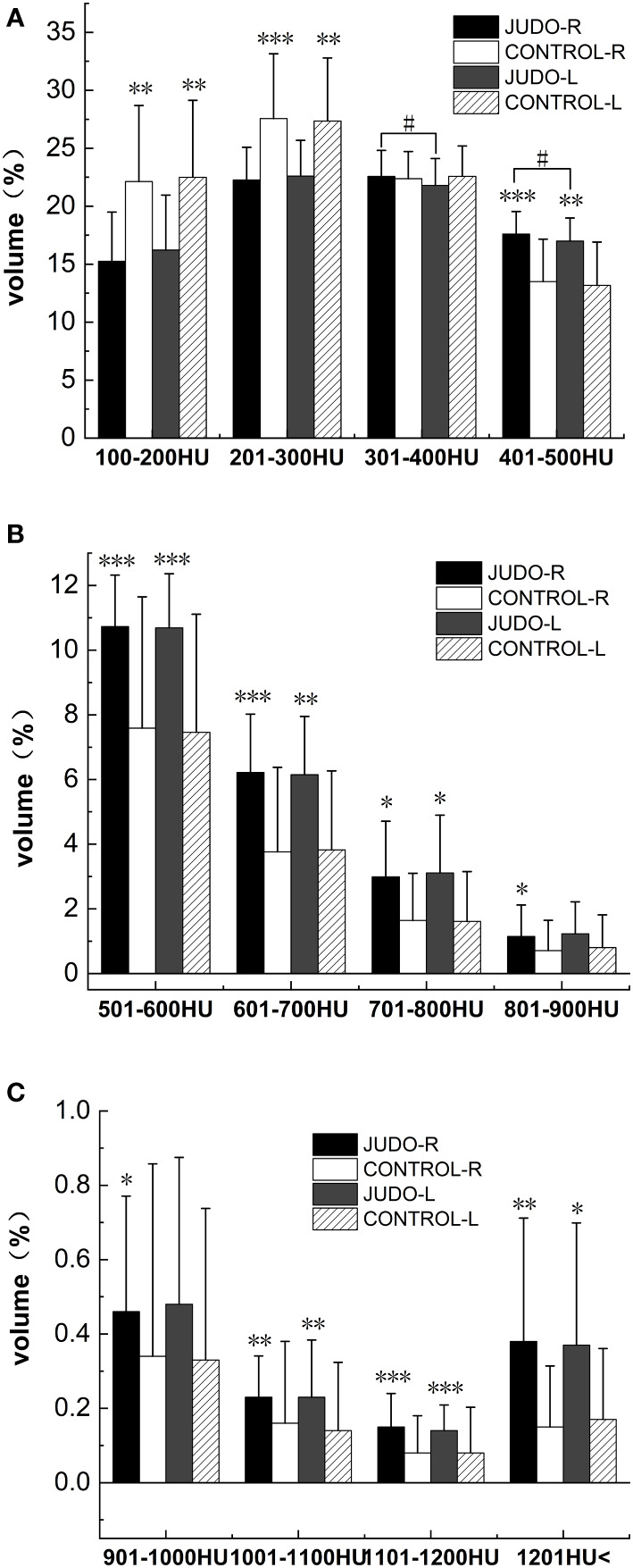
Comparison of %BTVi in the distal femur of subjects in both groups. **(A)** shows bone tissue volumes for low bone density in judo and control subjects; **(B)** shows bone tissue volumes for moderate bone density in judo and control subjects; **(C)** shows bone tissue volumes for high bone density in judo and control subjects (***, ** and * indicate statistically significant *P*-values of<0.001,<0.01 and<0.05, respectively, compared to control; # indicates the comparison of the judo athletes (the *P*-value with statistical significance is<0.05).

**Figure 5:**
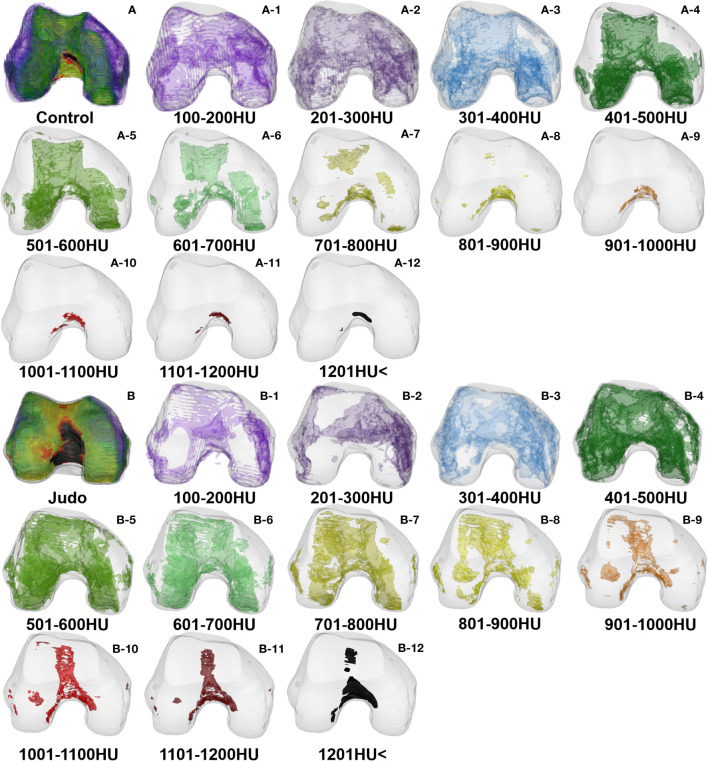
Bone tissue volume distribution of bone densities in the distal femur: **(A)** Control group **(A-1–A-12)** and **(B)** Judo group **(B-1–B-12)**.

The low (partial) bone density %BTVi of the distal femur was higher in the control group than in the judo athletes (*P*< 0.05). Low (partial), moderate and high bone density %BTVi of the distal femur were higher in judo athletes than in controls (*P*< 0.05), and low bone density %BTVi of the distal right femur was higher in judo athletes than in the distal left femur of judo (*P*< 0.05) (**Figures 4**, **5**; **Table 4**).

**Table 4:** Comparison of the bone tissue volume individuals in the distal femur subchondral bone between the groups (%).

Density classifications	Unit: HU	Right		*P* value	Left		*P* value
		JUDO	Control		JUDO	Control	
Low bone density	100-200	15.25 ± 4.246	22.13 ± 6.562	0.002	16.22 ± 4.732	22.49 ± 6.644	0.006
	201-300	22.27 ± 2.819	27.56 ± 5.594	0.001	22.60 ± 3.081	27.35 ± 5.422	0.002
	301-400	22.58 ± 2.238^#^	22.38 ± 2.342	0.819	21.79 ± 2.323	22.58 ± 2.613	0.394
	401-500	17.59 ± 1.960^#^	13.50 ± 3.657	0.001	16.99 ± 1.993	13.17 ± 3.736	0.002
Medium bone density	501-600	10.73 ± 1.589	7.59 ± 4.057	0.001	10.69 ± 1.674	7.46 ± 3.652	0.001
	601-700	6.22 ± 1.802	3.76 ± 2.619	0.001	6.15 ± 1.802	3.82 ± 2.449	0.004
	701-800	2.99 ± 1.716	1.64 ± 1.461	0.028	3.11 ± 1.783	1.61 ± 1.544	0.021
	801-900	1.15 ± 0.969	0.71 ± 0.935	0.033	1.23 ± 0.987	0.80 ± 1.012	0.056
High bone density	901-1000	0.46 ± 0.311	0.34 ± 0.518	0.041	0.48 ± 0.395	0.33 ± 0.408	0.05
	1001-1100	0.23 ± 0.111	0.16 ± 0.220	0.009	0.23 ± 0.154	0.14 ± 0.184	0.007
	1101-1200	0.15 ± 0.090	0.08 ± 0.100	0.001	0.14 ± 0.069	0.08 ± 0.123	0.001
	1201-maximum	0.38 ± 0.332	0.15 ± 0.164	0.002	0.37 ± 0.329	0.17 ± 0.191	0.016

# Indicate statistically significant P values of<.05 in the left and right %BTV for judo group, respectively.

**Figure 6:**
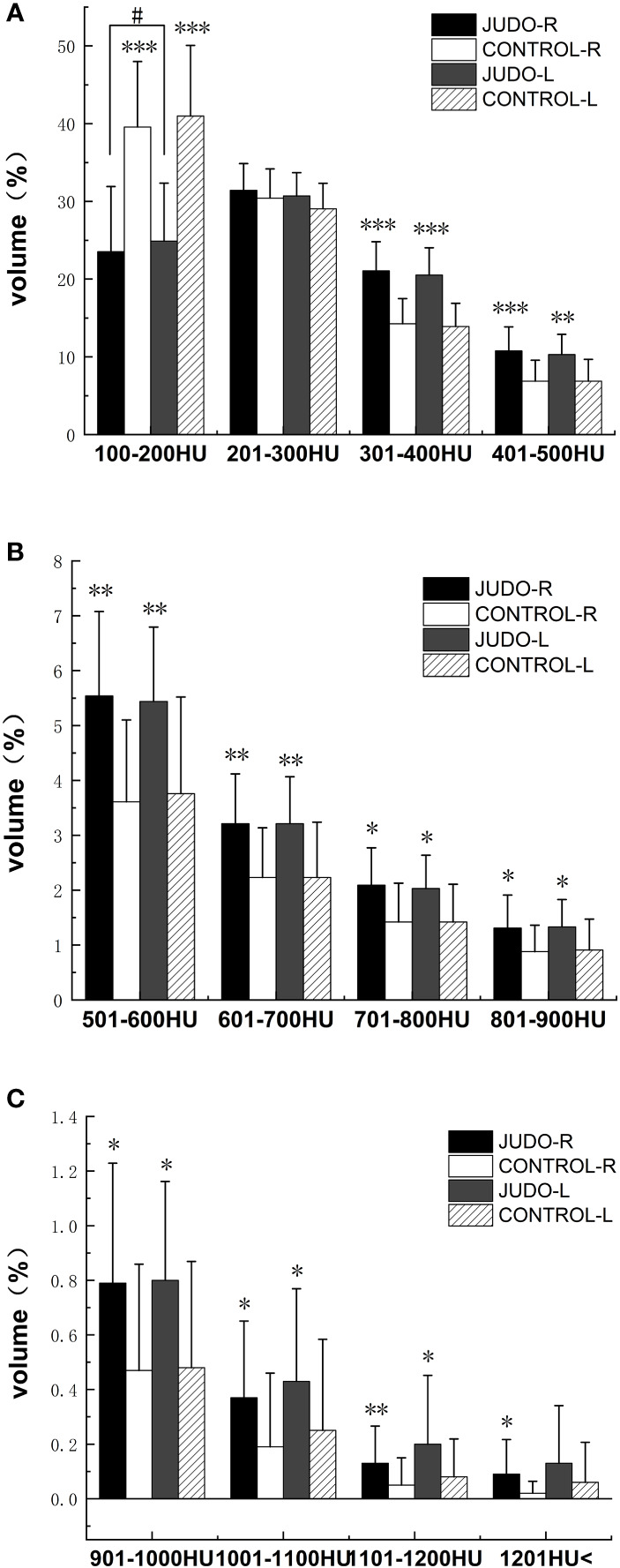
Comparison of %BTVi in the tibial plateau of subjects in both groups. **(A)** shows bone tissue volumes for low bone density in judo and control subjects; **(B)** shows bone tissue volumes for moderate bone density in judo and control subjects; **(C)** shows bone tissue volumes for high bone density in judo and control subjects (***, ** and * denote statistically significant P values < 0.001, < 0.01 and < 0.05 for control, respectively; # denotes P values for comparisons among judo athletes with statistical significance of < 0.05, respectively).

**Figure 7:**
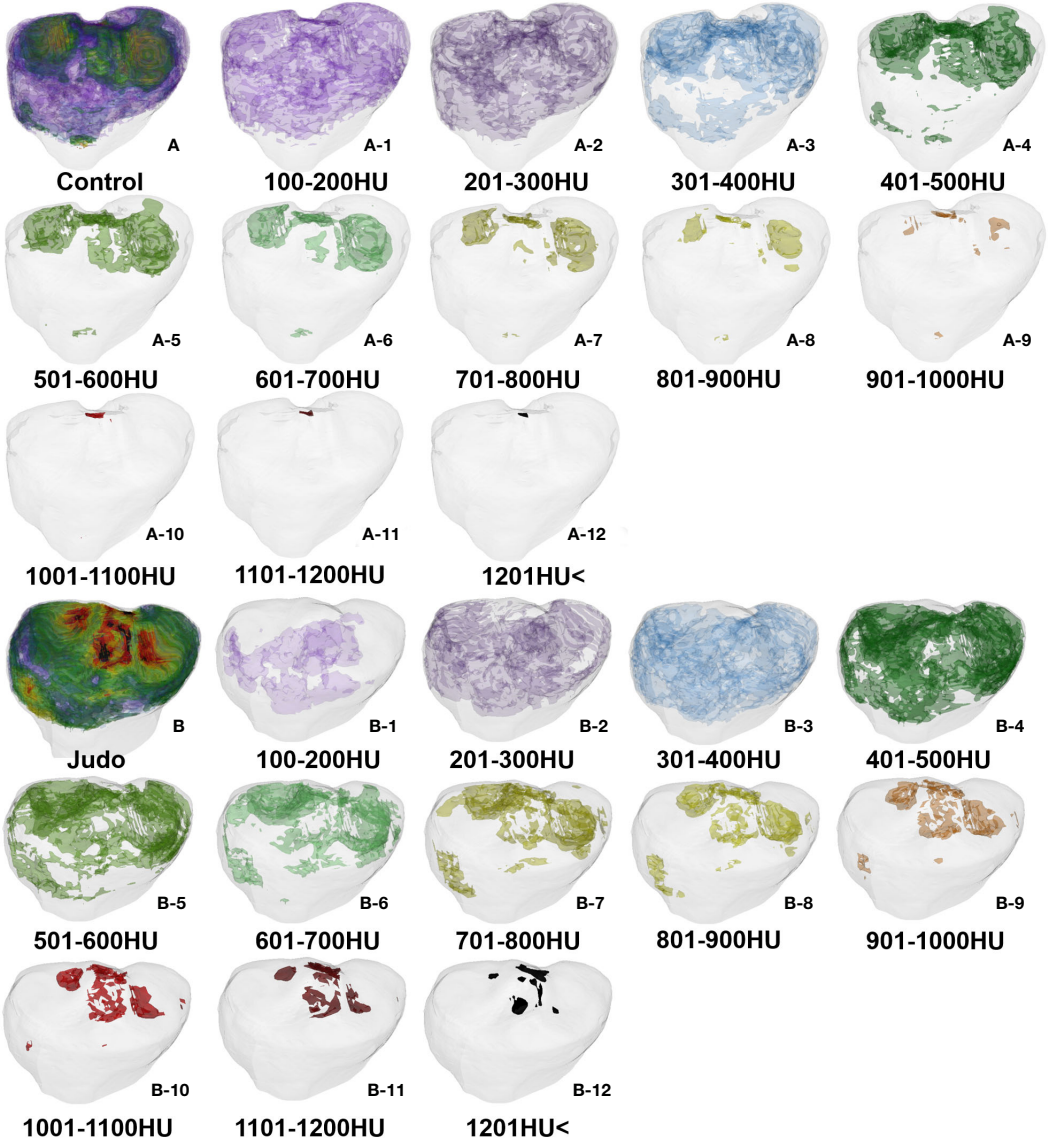
Bone tissue volume distribution of bone densities in the tibial plateau: **(A)** Control group **(A-1–A-12)** and **(B)** Judo group **(B-1–B-12)**.

Low bone density %BTVi within the tibial plateau was higher in all control groups than in judo athletes (*P*< 0.05). The low (partial), medium and high (partial) bone density %BTVi of the tibial plateau were higher in judo athletes than in controls (*P<* 0.05), and the low (partial) bone density %BTVi within the left tibial plateau was higher in judo athletes than in the right tibial plateau of judo (*P*< 0.05) (**Figures 6**, **7**; **Table 5**).

**Table 5:** Comparison of the bone tissue volume in the tibia plateau subchondral bone between the groups (%).

Density classifications	Unit: HU	Right		*P* value	Left		*P* value
		JUDO	Control		JUDO	Control	
Low bone density	100-200	23.54 ± 8.378	39.57 ± 8.434	0.001	24.90 ± 7.446^#^	40.98 ± 9.096	0.001
	201-300	31.11 ± 3.428	30.42 ± 3.766	0.604	30.71 ± 2.999	29.06 ± 3.276	0.162
	301-400	21.06 ± 3.769	14.27 ± 3.232	0.001	20.52 ± 3.510	13.90 ± 2.986	0.001
	401-500	10.76 ± 3.104	6.87 ± 2.703	0.001	10.30 ± 2.594	6.87 ± 2.808	0.002
Medium bone density	501-600	5.54 ± 1.538	3.61 ± 1.492	0.002	5.44 ± 1.353	3.76 ± 1.760	0.007
	601-700	3.21 ± 0.909	2.23 ± 0.905	0.006	3.21 ± 0.857	2.23 ± 1.008	0.008
	701-800	2.09 ± 0.682	1.42 ± 0.707	0.013	2.03 ± 0.605	1.42 ± 0.687	0.015
	801-900	1.31 ± 0.600	0.88 ± 0.481	0.041	1.33 ± 0.498	0.91 ± 0.563	0.042
High bone density	901-1000	0.79 ± 0.439	0.47 ± 0.389	0.045	0.80 ± 0.362	0.48 ± 0.389	0.026
	1001-1100	0.37 ± 0.281	0.19 ± 0.270	0.013	0.43 ± 0.339	0.25 ± 0.334	0.041
	1101-1200	0.13 ± 0.136	0.05 ± 0.100	0.005	0.20 ± 0.252	0.08 ± 0.139	0.029
	1201-maximum	0.09 ± 0.127	0.02 ± 0.044	0.021	0.13 ± 0.211	0.06 ± 0.147	0.081

# Indicate statistically significant P values of<.05 in the left and right %BTV for judo group, respectively.

## 4 Discussion

### 4.1 Differences in stress distribution at the knee joint surface between judo athletes and control subjects

The current study mainly found differences in the distribution of subchondral bone density (stress) on the knee facet between judo athletes and control subjects. The distal femur and tibial plateau had a larger area of high density in judo athletes than in the control group, and was predominantly distributed in zones 4, 5 and 6. Interestingly, this region is not in direct contact with the joint head and fovea, therefore we believe that the presence of ligamentous tissues starting and ending in this region plays a major role, such as the cruciate ligament of knee, meniscofemoral ligament. The anterior cruciate ligaments (ACL) originate from the anterior-medial aspect of the intercondylar eminence of the tibia and are oblique to the posterior superior lateral aspect, with fibers scalloped to the medial aspect of the lateral femoral condyle. The posterior cruciate ligaments (PCL) begin at the medial femoral condyle of the femoral intercondylar fossa and end at the posterior or posterior-inferior fossa of the proximal tibia. The ACL and PCL not only prevent anterior and posterior displacement of the tibia, but also limit joint movements such as hyperextension, flexion and rotation of the knee, thereby reducing the chance of knee dislocation. In particular, ACL injuries tend to occur during initial contact or shortly after non-contact (8), when the knee is often in an acutely variable valgus and internal/external rotation position (8–13).

According to the results of the subchondral bone tissue density distribution of the knee facets in both groups of subjects, the standing throwing technique (*Nage-waza*) and the ground grappling technique (*Katame-waza*) used in Olympic judo are the main factors that cause high-density zones in the 4th, 5th and 6th regions of the knee joint in judo athletes. Because of the large number of throwing techniques in judo, athletes often use combined upper-lower body throwing techniques such as *Osoto-gari*, *Kouchi-gari*, and *Seoi-nage*. While in the phase of holding, judo player and opponents (*uke*) first use upper limb movements to instantly destroy the opponent’s upper limb and torso movements; then they use unilateral or bilateral lower limb knee flexion and extension movements to lower the body’s center of gravity and throw the opponent to the ground. Although these movements are fast and explosive, the judo athlete still goes through a full flexion process when performing throwing techniques - the knee goes from a slightly flexed position to a quarter flexion to a half-flexed position, while the tension and pull of the ACL increases with the change in joint angle. As a result, the 4th, 5th and 6th regions of the judo athlete show varying degrees of high-density distribution. Early studies found that black belt judo athletes were able to maximize the angular velocity of the knee and ankle joints of the sweeping leg when using the *osoto-gari* and *osoto-otoshi* movements. The sweeping leg was used to hook the *uke* lower extremity for the purpose of destabilizing the opponent’s limb. In contrast, novice judo groups have difficulty executing a mature and complete knee flexion movement before the sweeping leg action contact and immobilize the opponent’s lower extremity (14). The knee flexion action in judo not only assumes the role of lowering one’s own center of gravity to enhance one’s stability but also assumes an aggressive action with the purpose of destabilizing the opponent’s lower limb, thereby increasing the overall chances of successful throwing action. During the London Olympics, 54.7% of judo matches were won using the throwing technique, followed by the penalty technique (21.7%), stepping out of the playing area, or the grappling technique (16.5%) (15). Early epidemiological studies (2, 16) found that during both throwing and being thrown, judo athletes had a prevalence of 28% in knee injuries. Among these injuries, ACL rupture was about 5.6% and also often accompanied by medial collateral ligament injuries, while *Ouchi-gari, Osoto-otoshi, Seoi-nage, and Osoto-gari* were common technical movements that caused knee injuries in judo athletes. It also caused knee flexion-external rotation movements, which lay an important potential risk for injury to the medial collateral ligament and ACL of the knee.

Current research has also focused on the effects of lower extremity muscle strength on the cruciate ligament. For example, how does knee flexor (KF) strength affect lower extremity knee extensor (KE)strength. The imbalance between KF and KE groups has been suggested as a potential mechanism for increasing lower extremity injuries such as ACL tears (17, 18). Weight-bearing running requires greater knee and ankle extensor strength, with males in particular exhibiting greater muscular strength in the knee than females (19). However, sports medicine practitioners and sports biomechanics believe that the interaction between the KF and KE muscle groups determines the degree of tibial plateau displacement and ACL injury. It has also been reported that unilateral lower extremity muscular fatigue in judo athletes adversely affects the bipedal standing balance posture, but the adverse effects are more pronounced when the eyes are closed, especially when the muscle fatigue developed by unilateral knee flexion-extension affects the ability to perform bilateral limb movements (20). The deformation of sports technique caused by sports fatigue is one of the main factors leading to injuries in athletes. It is well known that ligamentous tissue is unable to generate stress, while muscle tissue, due to its unique muscle fiber contraction properties, causes movement of the joint resulting in tension. Tension and stress transfer in the ligament, while the change in tension magnitude varies with the magnitude of joint movement. An association has been found between lower extremity jumping and landing injuries and lower extremity overuse injuries. During repeated jumping and landing movements, the forward and lateral displacement movements generated by the knee joint of the lower extremity added additional impact and tension to the musculature and skeletal structures of the lower extremity (21).

When the joint is in the over flexion-over extension state, the fibrous connective tissue in the ligaments tears, which eventually leads to ligamentous tissue rupture. In the subchondral bone area of the knee facet of the judo group, we noted different degrees of high-density tissue distribution in the ACL and PCL starting and stopping points of the knee joint in judo subjects. This demonstrates that the knee flexion technique in judo may cause overload tension in the cruciate ligaments, and laterally suggests that the technical characteristics of judo are potential factors contributing to cruciate ligament injuries in the knee. Especially in judo training, club managers and team coaches should pay attention to the accuracy, rationality and science of the technique, especially to the fatigue level of the knee muscle groups and the stability level of the knee joint of the athletes. It is recommended to include daily fatigue testing of the muscle groups around the knee joint, knee stability testing and epidemiological education on the mechanisms of knee sports injuries to prevent knee injuries in judo athletes during daily training.

In general, from a functional anatomical point of view, the knee joint is protected by the surrounding ligaments and joint capsule, and the knee joint is primarily responsible for more upright trunk (weight-bearing function), substantial flexion-extension and very limited inversion-extension, internal-external panning, and internal and external rotation of the tibia. From a kinematic point of view, the knee joint move against each other in the form of compression, collision, and friction, which may result in a significant contact stress distribution on both joint surfaces. Based on the results of the current study, we found no significant distribution of large area of high-density areas on the distal trochlear surface of the femur (except zones 4, 5, and 6) in judo athletes and control subjects. The distribution of high-density areas in the subchondral bone of the articular surface of the tibial plateau was concentrated in regions 1, 2, 3, 4, 5, 6, 7 and 8 in judo athletes. The control subjects (very few) were concentrated in the 2nd, 4th, 5th, and 7th regions, and some control subjects had the distribution of large areas in the medial or lateral compartment of the tibial plateau (most control subjects had lower levels of subchondral bone tissue density). This suggests that the medial-lateral interval of the knee joint in judo athletes may be stimulated by different levels of stress loading from knee flexion-extension, self-gravity, opponent’s weight, and technical movement patterns, resulting in a distribution of high-density areas of varying size in the medial-lateral interval. In contrast, the medial-lateral interval of the tibial plateau in control subjects was loaded by their own gravity and showed small areas of high density or appeared in the medial or lateral interval of the tibial plateau. Because the distribution of high-density areas in the medial region of the tibial plateau was seen in both groups of subjects, the results were more similar to those reported in earlier osteoarthritis of the knee (KOA). That is, the stress stimulation of the lower extremity knee joint may first lead to a high-density distribution of the medial space of the tibial plateau. One study performed MRI measurements of local knee cartilage strains in normal healthy knees in response to dynamic jumping activity and found significant cartilage strains in the medial and lateral intercompartmental compartments of the tibial plateau, with an average decrease of 5% in each compartment. However, these strains varied with the position of the distal femur within the intercompartmental compartment, with the maximum compressive strain reached 8% in the medial plateau and 7% in the lateral plateau. In addition, localized areas of cartilage of the medial-lateral femoral condyle also showed significant compressive strains, reaching maxima of 6% and 3%, respectively (22). This study found that the medial-lateral femoral condylar glides also produced varying degrees of strain. However, interestingly, we did not find significant density size changes and asymmetrical joint space in the distal femur (medial and lateral femoral trochlea) in both groups of subjects, while the results of cartilage strain in the medial-lateral intertrochanteric compartment of the tibial plateau were more consistent with the results of high-density distribution in the tibial plateau (medial-lateral) in this study. The cartilage and subchondral bone strains within the knee joint reflect the presence of benign stress distribution and transmission within the knee joint, as unbalanced medial-lateral knee loading is also often considered to be an important factor in the development and progression of KOA.

A summary of the effects of the half squat technique and quarter squat on the lower extremity joints found that for knee flexion angles exceeding 50° in the deep squat, knee stress loads were in the acceptable range. If a cadaveric knee specimen is flexed at 90°, compressive forces and stresses occur within the patellofemoral joint. As the knee flexion angle increases, the wrapping effect contributes to enhanced load distribution and enhanced force transfer (23). The results of this study are consistent with the findings of this paper, where high-density areas of varying size were observed on the patellofemoral joint surface of the knee in judo athletes (region 4; approximately 12 individuals; 24 knees), while some control subjects (region 4; approximately 6 individuals; 12 knees) showed a distribution of high-density areas on the patellofemoral joint surface. This suggests that quadriceps tension increases with increasing knee joint angle and that the patellar ligament anchors the patella to the patellofemoral joint surface, increasing the compressive stress on the patellofemoral joint surface by the patella. In general, healthy tissues are significantly more capable of withstanding and adapting to loads than pathological tissues; the structure of soft tissues is significantly better in younger than in older adults. This change in tissue biology might be the result of the interaction between the level of load generated by a specific movement and a specific structure (24–26). However, immediate changes in articular cartilage morphology do not adversely affect cartilage morphological structure or composition. Interestingly, some studies have reported elevated cartilage content of the medial knee and patellofemoral articular surfaces after 3 months of marathon exercise (27). One study found elevated T2-relaxation values within the articular cartilage of the knee after marathon exercise in a high BMI population, presumably as a result of changes in biochemical indicators of articular cartilage (28). However, it has also been found that such tissues do not undergo morphological changes (29). The fact that knee cartilage can recover its structural and functional properties after being subjected to running-related loads reflects the fact that articular cartilage can withstand higher loads and repeated stimulation after short periods of rest. Indeed, repeated exposure to mechanical loads within the safe physiological range that cartilage can tolerate may trigger a positive adaptive response (30). Subchondral bone tissue can both buffer intra-articular stress transmission and have a range of material strength and stiffness (31). Although the density and volume percentage of subchondral bone tissue in athletes reflect certain mechanical force sensitivity and bone remodeling properties, we still recommend regular tracking of the occurrence of abnormal stress distribution in the joints of judo athletes, especially during training. Exam to assess knee injury risk and recovery of limb function.

### 4.2 Differences in the distribution of subchondral bone remodeling in knee joints promoted by mechanical stress in judo

In axial, sagittal and coronal section images, the subchondral bone sections of the knee in judo athletes showed a concentric distribution with higher density in the ligament initiation and termination areas, osteoarticular contact areas and the epiphyseal line areas. In contrast, the control group showed a laminar distribution with lower density in the osteoarticular contact region and even lower density in the non-direct contact region. Compared to the control group, judo athletes had higher reserves of low (partial), medium and high %BTVi of subchondral bone in the knee ( proximal femur and tibia plateau). This indicates that long-term judo training can enhance the formation of subchondral bone tissue and bone remodeling level in the knee joint of judo athletes. Given the technical characteristics of judo sports, the location of knee bone remodeling in judo athletes is an important factor in addition to the location of direct articular cartilage contact, and the height of bone remodeling at the starting and ending points of the ligaments within the knee joint. We observed a small distribution of bone tissue of low bone density in areas other than the cartilage-covered area of the knee joint in judo athletes, such as the anterior edge of the tibial plateau and the edge of the lateral compartment. In contrast, the presence of low-density bone tissue in the knee joint of control subjects was around regions outside the articular cartilage envelope, such as the inner region of the distal femoral and regions 1 and 9. Therefore, based on the density distribution in the control subjects we concluded that the normal areas of stress within the knee joint were the distal femoral trochanteric and patellofemoral articular surfaces (partially) and part of the medial and lateral interventricular regions of the tibial plateau (regions 2, 3, 4, 5, partial 6, 7, and 8).

In addition, we noted that the distribution of high-density areas in the region of the medial-lateral tibial plateau in judo athletes was not symmetrically distributed (2nd, 3rd-8th, 9th or 1st, 2nd-7th, 8th), but showed an anterior-posterior oblique symmetrical distribution (e.g., 7th, 8th-2nd, 3rd). Although individual control subjects showed high density areas in the region of the medial-lateral tibial plateau with oblique symmetric distribution, most control subjects showed occasional high-density areas in the medial-lateral tibial plateau (2nd or 3rd, 7th or 8th). This suggests that the factors contributing to the distribution of anterior-posterior symmetric high-density areas in the medial-lateral tibial plateau in judo athletes are determined by the specificity of the technique of judo. Judo is an exercise containing a high number of micro-flexions, quarter-flexion, and knee hemi-flexion movements. While increasing the tension and pulling force of the cruciate ligament, the medial-lateral tibial plateau is instantaneously subjected to the pressure load generated by its own gravity, the weight of the opponent and the muscles around the knee joint. Such pressure distribution results in a special distribution of high-density areas in the subchondral bone of the medial-lateral interval of the knee joint even after the meniscus has absorbed most of the stress. In addition, because of the insufficient awareness of judo athlete regarding the standardized use of throwing (Nage-waza), foot technique (Ashi-Waza) or joint technique (Kansetsu-Waza) during competition and training, the knee joint performs a flexion movement with varying degrees of valgus and tibial rotation movements. This leads to an anterior-posterior oblique symmetrical distribution of the high-density zone of the medial-lateral tibial plateau in judo athletes, which also seriously affects cruciate ligament safety. The long-term mechanical stress transmitted in the joint leads to long-term adaptive changes in the density and mineral salt content of the bone matrix, indicating the load history of the articular subchondral bone under long-term stress (6). The location of the high-density area is used to judge whether the area is in line with normal Intra-articular stress distribution, by correcting the technical movements of judo athletes, reducing the force in the unreasonable area of the joint, thereby reducing the cumulative injury of the athlete’s knee joint. In the knee joint, the subchondral bone is closely connected with the articular cartilage (32, 33), and healthy and non-abrasive articular cartilage attaches to the subchondral bone and provides a stable mechanical joint support for the subchondral bone and maintains homeostasis metabolism (34–36).

According to Wolff’s law (37, 38), stress factors such as compressive stress, tensile force, shear force, and hydrostatic pressure generated by long-term sports or body movements may lead to remodeling or resorption changes in the subchondral bone of the articular cartilage, reflecting bone the adaptive characteristics of organizations (39). It can be seen from the above that the bone density will change with the stress distribution in the articular cartilage, thereby increasing the probability of damage to the indirect stress zone in the joint. But we sports medicine scientists understand the ornamental and competitive nature of combat sports. Athletes (e.g., judo, taekwondo, boxing, karate) beat their opponents under the rules of the sport, although we can reduce the percentage of injuries in daily training to a certain extent. However, due to the competitive characteristics of athletes, accidents and safety issues of technical application may not be considered during the competition. Although moderate mechanical loading of the joint helps to maintain the integrity of the articular cartilage, joint inactivity or overuse may lead to cartilage degeneration (36, 40, 41), and modern biomechanics and materials science have further investigated new sports equipment and protective gear, But the high protective performance of sports equipment is too comfortable, reducing the adaptive stress load of the joints, and to a certain extent, will it also increase non-sports technical injuries? In this case, further research is required. Since any tissue is limited by its tissue physiology and physical properties, once the external load exceeds the yield limit of that tissue, tearing and breaking.

## 5 Limitations

At present, the research has the following limitations, 1) kinematic and biomechanical experiments on judo exercise techniques were not performed in this study; 2) only current imaging data of judo athletes were obtained in this study, but no imaging data were obtained prior to participation in judo; 3) the study did not determine which direct factors contributed to the high-density areas in the knee joints of judo athletes.

## 6 Conclusions

The results of this study showed that judo athletes exhibited high bone density distribution in the femoral intercondylar fossa, the patellofemoral articular surface, the medial and lateral compartments of the tibial plateau, and the tibial intercondylar eminence. The reasons for the distribution of large areas of high bone density in the articular surfaces of the distal femur and the tibial plateau in judo athletes, and the remodeling of the subchondral bone tissue in the distal femur and the tibial plateau were demonstrated. The synergistic effect of intra-articular stress transmission and load may be caused by factors such as long-term knee flexion self-bearing, judo lower body technique, training years and competition frequency, and puberty development. In judo athletes, the transition pattern of bone tissue of the distal femur and tibia plateau is from low-density to medium-density bone. However, a small amount of medium-density bone tissue was transformed into high-density bone tissue, and only in the stressed area, while a small amount of low-density bone remained in the non-stressed area.

## Data availability statement

The raw data supporting the conclusions of this article will be made available by the authors, without undue reservation.

## Ethics statement

The studies involving human participants were reviewed and approved by Biological and Medical Ethics Committee, North University of China. The patients/participants provided their written informed consent to participate in this study.

## Author contributions

GX and ZL completed the experiment and wrote the manuscript. ZL and GX directed the design test and repaired the manuscript. ZL provided research information. QW and CL edited the article. DC and LW provided experimental and publication funding. The rest of the authors put forward valuable opinions on the whole subject design. All authors contributed to the article and approved the submitted version.

## References

[1] Messner N. Judo celebrates the planet (2019). Available at: https://www.ijf.org/news/show/judo-celebrates-the-planet.

[2] Pocecco E Ruedl G Stankovic N Sterkowicz S Del Vecchio FB Gutiérrez-García C. Injuries in judo: A systematic literature review including suggestions for prevention. Br J Sports Med (2013) 47:1139–43. doi: 10.1136/bjsports-2013-092886 24255909

[3] Wojciech J Cynarski MK. Injuries in martial arts and combat sports – A comparative study. Arch BUDO (2008) 4:91–7.

[4] Frey A Müller W. Heberden-Arthrosen bei Judo-Sportlern [Heberden arthroses in judo athletes]. Schweiz Med Wochenschr. (1984) 114(2):40–7. 6701483

[5] Strasser P Hauser M Häuselmann HJ Michel BA Frei A Stucki G. Traumatic finger polyarthrosis in judo athletes: A follow-up study. Z Rheumatol (1997) 56(6):342–50. doi: 10.1007/s003930050048 9487650

[6] Eckstein F Muller-Gerbl M Steinlechner M Kierse R Putz R. Subchondral bone density in the human elbow assessed by computed tomography osteoabsorptiometry: A reflection of the loading history of the joint surfaces. J Orthop Res (1995) 13:268–78. doi: 10.1002/jor.1100130215 7722764

[7] Xu G Liu H Zhang L. Characterization of changes in subchondral bone tissue density of the ankle joint in taekwondo players. Front Bioeng Biotechnol (2022) 10. doi: 10.3389/fbioe.2022.872258 PMC9114634 35600898

[8] Krosshaug T Nakamae A Boden BP Engebretsen L Smith G Slauterbeck JR. Mechanisms of anterior cruciate ligament injury in basketball. Am J Sports Med (2007) 35:359–67. doi: 10.1177/0363546506293899 17092928

[9] Boden BP Dean GS Feagin JA Garrett WE. Mechanisms of anterior cruciate ligament injury. Orthopedics (2000) 23(6):573–8. doi: 10.3928/0147-7447-20000601-15 10875418

[10] Boden BP Torg JS Knowles SB Hewett TE. Video analysis of anterior cruciate ligament injury: Abnormalities in hip and ankle kinematics. Am J Sports Med (2009) 37(2):252–9. doi: 10.1177/0363546508328107 19182110

[11] Hewett TE Torg JS Boden BP. Video analysis of trunk and knee motion during non-contact anterior cruciate ligament injury in female athletes: Lateral trunk and knee abduction motion are combined components of the injury mechanism. Br J Sports Med (2009) 43:417–22. doi: 10.1136/bjsm.2009.059162 PMC4062295 19372088

[12] Olsen O Myklebust G Engebretsen L Bahr R. Injury mechanisms for anterior cruciate ligament injuries in team handball. Am J Sports Med (2004) 32:1002–12. doi: 10.1177/0363546503261724 15150050

[13] Koga H Nakamae A Shima Y Iwasa J Myklebust G Engebretsen L. Mechanisms for noncontact anterior cruciate ligament injuries: Knee joint kinematics in 10 injury situations from female team handball and basketball. Am J sports Med (2010) 38:2218–25. doi: 10.1177/0363546510373570 20595545

[14] Imamura R Johnson B. A kinematic analysis of a judo leg sweep: major outer leg reap–osoto-gari. Sports Biomech (2003) 2:191–201. doi: 10.1080/14763140308522817 14737927

[15] Ishii T Ae M Suzuki Y Kobayashi Y. Kinematic comparison of the seoi-nage judo technique between elite and college athletes. Sports Biomech (2018) 17:238–50. doi: 10.1080/14763141.2017.1284256 28632049

[16] Petrisor BA Del Fabbro G Madden K Khan M Joslin J Bhandari M. Injury in Brazilian jiu-jitsu training. Sports Health: A Multidiscip Approach (2019) 11:432–9. doi: 10.1177/1941738119849112 PMC6745816 31173700

[17] Read PJ Oliver JL De Ste Croix MBA Myer GD Lloyd RS. Neuromuscular risk factors for knee and ankle ligament injuries in Male youth soccer players. Sports Med (2016) 46:1059–66. doi: 10.1007/s40279-016-0479-z PMC5501175 26856339

[18] El-Ashker S Carson BP Ayala F De Ste Croix M. Sex-related differences in joint-angle-specific functional hamstring-to-quadriceps strength ratios. Knee Surg Sports Traumatol Arthrosc (2017) 25:949–57. doi: 10.1007/s00167-015-3684-7 26149462

[19] Wagers KD Lobb NJ Fain AC Seymore KD Brown TN. Sex impact on knee and ankle muscle extensor forces during loaded running. Biomechanics (2022) 2:421–30. doi: 10.3390/biomechanics2030032

[20] Ghram A Young JD Soori R Behm DG. Unilateral knee and ankle joint fatigue induce similar impairment to bipedal balance in judo athletes. J Hum Kinet (2019) 66:7–18. doi: 10.2478/hukin-2018-0063 30988836 PMC6458584

[21] De Bleecker C Vermeulen S De Blaiser C Willems T De Ridder R Roosen P. Relationship between jump-landing kinematics and lower extremity overuse injuries in physically active populations: A systematic review and meta-analysis. Sports Med (2020) 50:1515–32. doi: 10.1007/s40279-020-01296-7 32514700

[22] Sutter EG Widmyer MR Utturkar GM Spritzer CE Garrett WE DeFrate LE. *In vivo* measurement of localized tibiofemoral cartilage strains in response to dynamic activity. Am J Sports Med (2015) 43:370–6. doi: 10.1177/0363546514559821 PMC4315145 25504809

[23] Hartmann H Wirth K Klusemann M. Analysis of the load on the knee joint and vertebral column with changes in squatting depth and weight load. Sports Med (2013) 43:993–1008. doi: 10.1007/s40279-013-0073-6 23821469

[24] McCarthy MM Hannafin JA. The mature athlete: Aging tendon and ligament. Sports Health (2014) 6:41–8. doi: 10.1177/1941738113485691 PMC3874221 24427441

[25] Dye SF. The knee as a biologic transmission with an envelope of function: a theory. Clin Orthop Relat Res (1996) 325:10–8. doi: 10.1097/00003086-199604000-00003 8998861

[26] Gabbett T Sancho I Dingenen B Willy RW. When progressing training loads, what are the considerations for healthy and injured athletes? Br J Sports Med (2021) 55:947–8. doi: 10.1136/bjsports-2020-103769 33837004

[27] Luke AC Stehling C Stahl R Li X Kay T Takamoto S. High-field magnetic resonance imaging assessment of articular cartilage before and after marathon running: Does long-distance running lead to cartilage damage? Am J sports Med (2010) 38:2273–80. doi: 10.1177/0363546510372799 20631252

[28] Wang Z Ai S Tian F Liow MHL Wang S Zhao J. Higher body mass index is associated with biochemical changes in knee articular cartilage after marathon running: A quantitative T2-relaxation MRI study. Orthopaedic J Sports Med (2020) 8:232596712094387. doi: 10.1177/2325967120943874 PMC7427140 32851106

[29] Nathani A Gold GE Monu U Hargreaves B Finlay AK Rubin EB. Does injection of hyaluronic acid protect against early cartilage injury seen after marathon running? A randomized controlled trial utilizing high-field magnetic resonance imaging. Am J Sports Med (2019) 47:3414–22. doi: 10.1177/0363546519879138 31634003

[30] Khan MCM Donovan J O Charlton JM Roy J Hunt MA Esculier J. The influence of running on lower limb cartilage: A systematic review and meta-analysis. Sports Med (2022) 52:55–74. doi: 10.1007/s40279-021-01533-7 34478109

[31] Zhen G Cao X. Targeting TGFβ signaling in subchondral bone and articular cartilage homeostasis. Trends Pharmacol Sci (2014) 35:227–36. doi: 10.1016/j.tips.2014.03.005 PMC4058854 24745631

[32] Goldring MB Goldring SR. Articular cartilage and subchondral bone in the pathogenesis of osteoarthritis. Ann New York Acad Sci (2010) 1192:230–7. doi: 10.1111/j.1749-6632.2009.05240.x 20392241

[33] Barr AJ Campbell TM Hopkinson D Kingsbury SR Bowes MA Conaghan PG. A systematic review of the relationship between subchondral bone features, pain and structural pathology in peripheral joint osteoarthritis. Arthritis Res Ther (2015) 17:228. doi: 10.1186/s13075-015-0735-x 26303219 PMC4548899

[34] Donell S. Subchondral bone remodelling in osteoarthritis. EFORT Open Rev (2019) 4:221–9. doi: 10.1302/2058-5241.4.180102 PMC6549114 31210964

[35] Li G Yin J Gao J Cheng TS Pavlos NJ Zhang C. Subchondral bone in osteoarthritis: Insight into risk factors and microstructural changes. Arthritis Res Ther (2013) 15:223–3. doi: 10.1186/ar4405 PMC4061721 24321104

[36] Castañeda S Roman-Blas JA Largo R Herrero-Beaumont G. Subchondral bone as a key target for osteoarthritis treatment. Biochem Pharmacol (2012) 83:315–23. doi: 10.1016/j.bcp.2011.09.018 21964345

[37] Teichtahl AJ Wluka AE Wijethilake P Wang Y Ghasem-Zadeh A Cicuttini FM. Wolff’s law in action: A mechanism for early knee osteoarthritis. Arthritis Res Ther (2015) 17(1):207. doi: 10.1186/s13075-015-0738-7 26324398 PMC4556408

[38] Wolff J. The classic: on the inner architecture of bones and its importance for bone growth. 1870. Clin Orthop Relat Res (2010) 468:1056–65. doi: 10.1007/s11999-010-1239-2 PMC2835576 20162387

[39] Lara B Salinero JJ Gutiérrez J Areces F Abián-Vicén J Ruiz-Vicente D. Influence of endurance running on calcaneal bone stiffness in male and female runners. Eur J Appl Physiol (2016) 116:327–33. doi: 10.1007/s00421-015-3285-7 26520837

[40] Campbell TM Reilly K Laneuville O Uhthoff H Trudel G. Bone replaces articular cartilage in the rat knee joint after prolonged immobilization. Bone (2018) 106:42–51. doi: 10.1016/j.bone.2017.09.018 28974461

[41] Nomura M Sakitani N Iwasawa H Kohara Y Takano S Wakimoto Y. Thinning of articular cartilage after joint unloading or immobilization. an experimental investigation of the pathogenesis in mice. Osteoarthr Cartil (2017) 25:727–36. doi: 10.1016/j.joca.2016.11.013 27916560

