# Supplementary material for: Variation characteristics of stress distribution in the subchondral bone of the knee joint of judo athletes with long-term stress changes
Source: Front Endocrinol (Lausanne). 2023 Jan 24;13:1082799. doi: 10.3389/fendo.2022.1082799 (PMC9909959; doi:10.3389/fendo.2022.1082799)
Supplement: Supplementary file 1 [file DataSheet_1.zip › distribution pattern of high density area in knee.pdf]

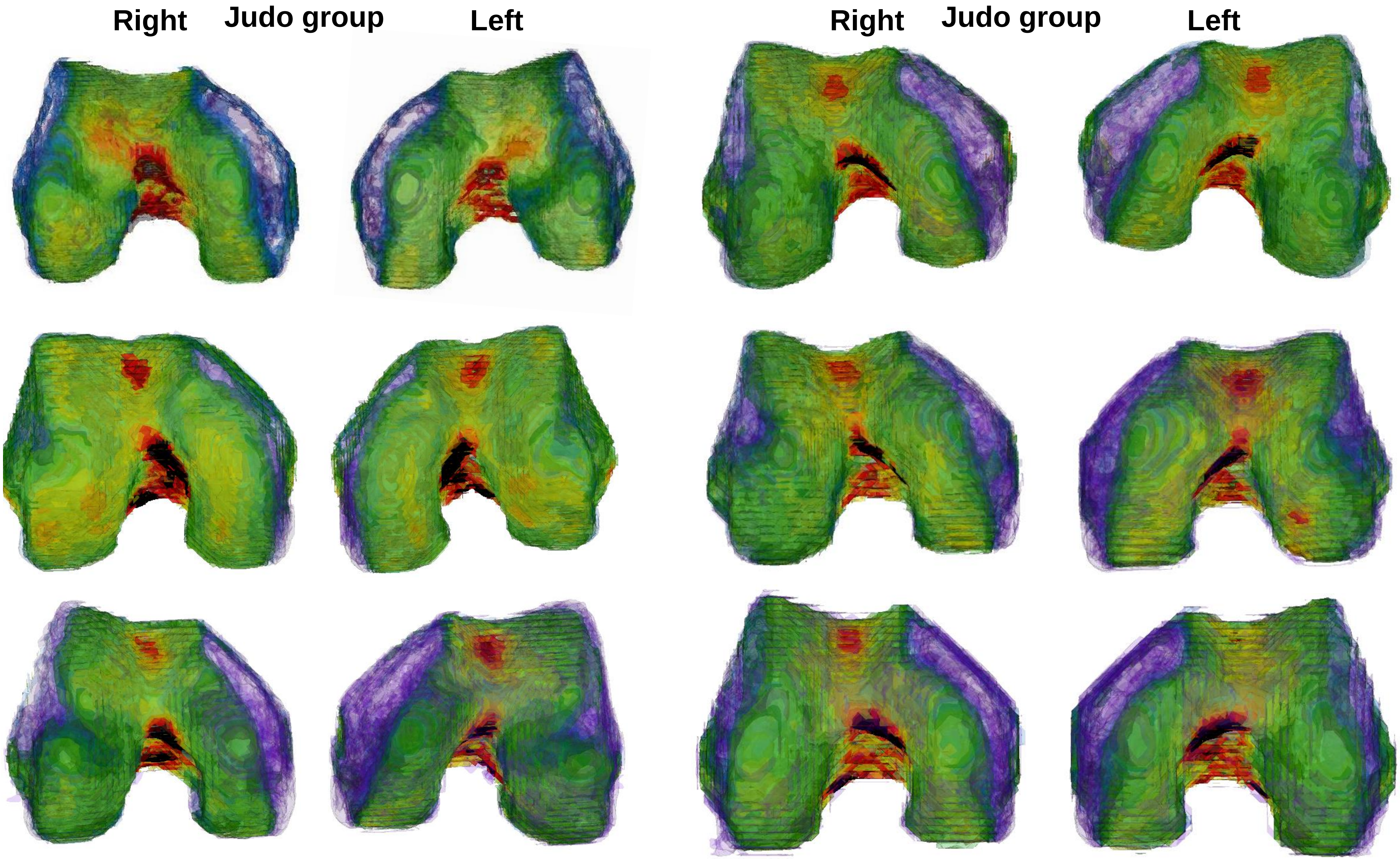
Right
Judo group
Left
Right
Judo group
Left

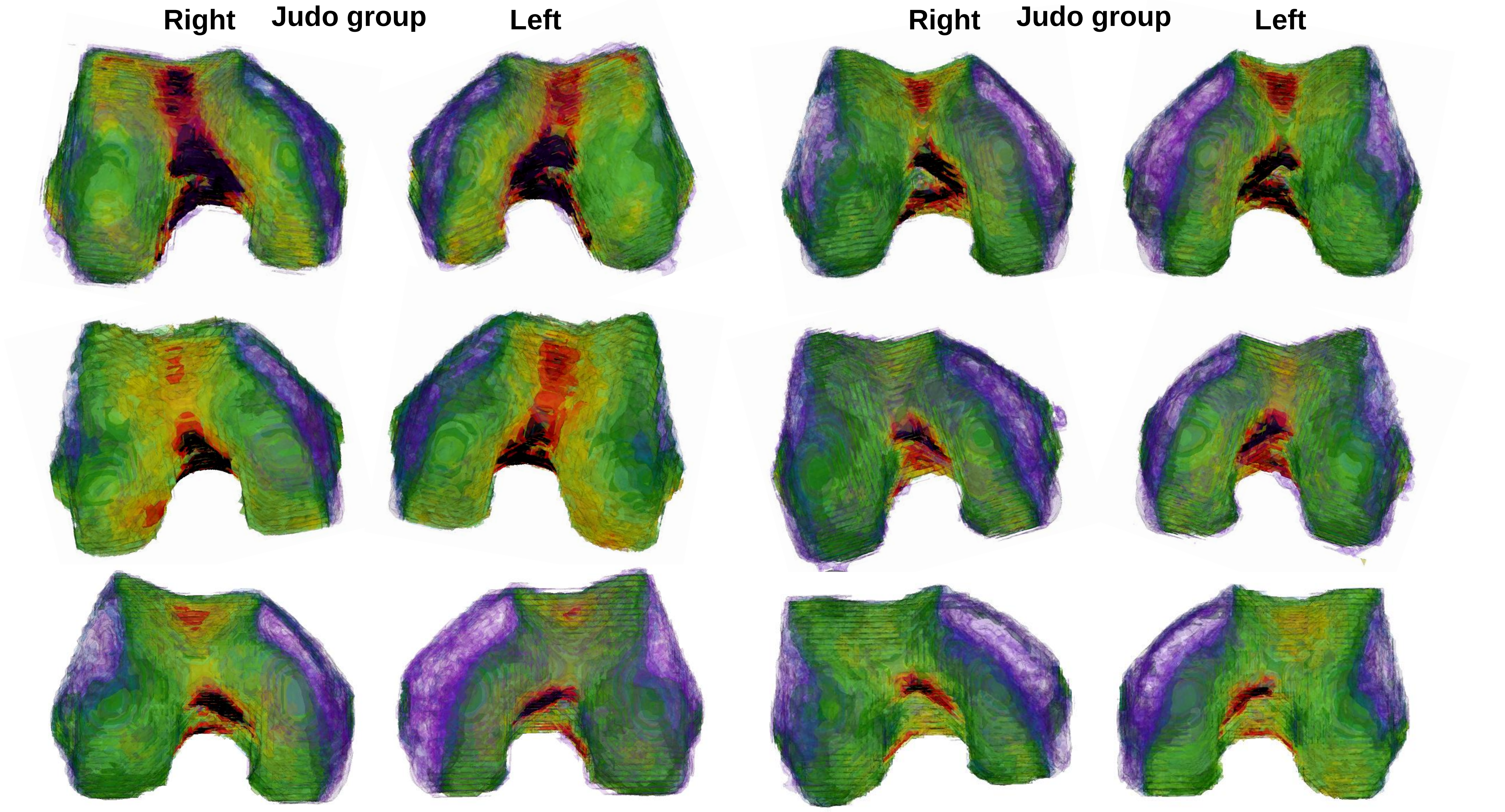
Right
Judo group
Left
Right
Judo group
Left

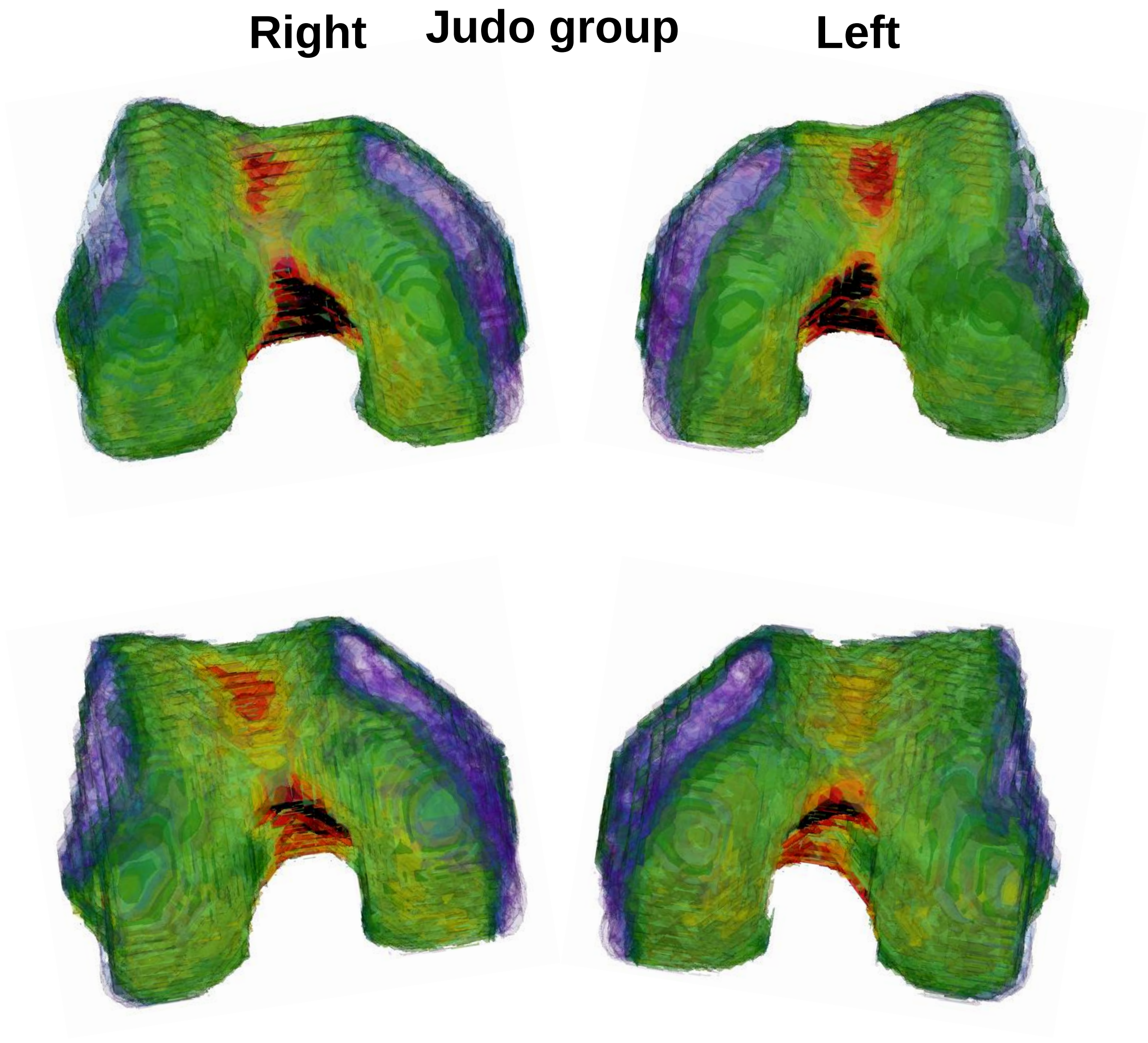
Right
Judo group
Left

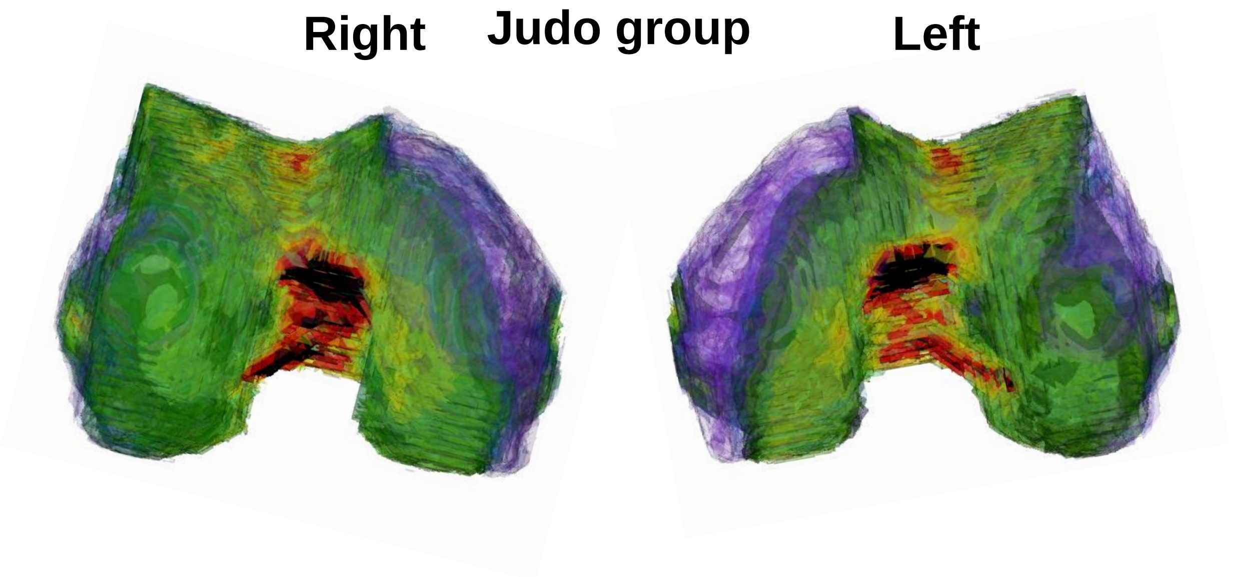
Right
Judo group
Left

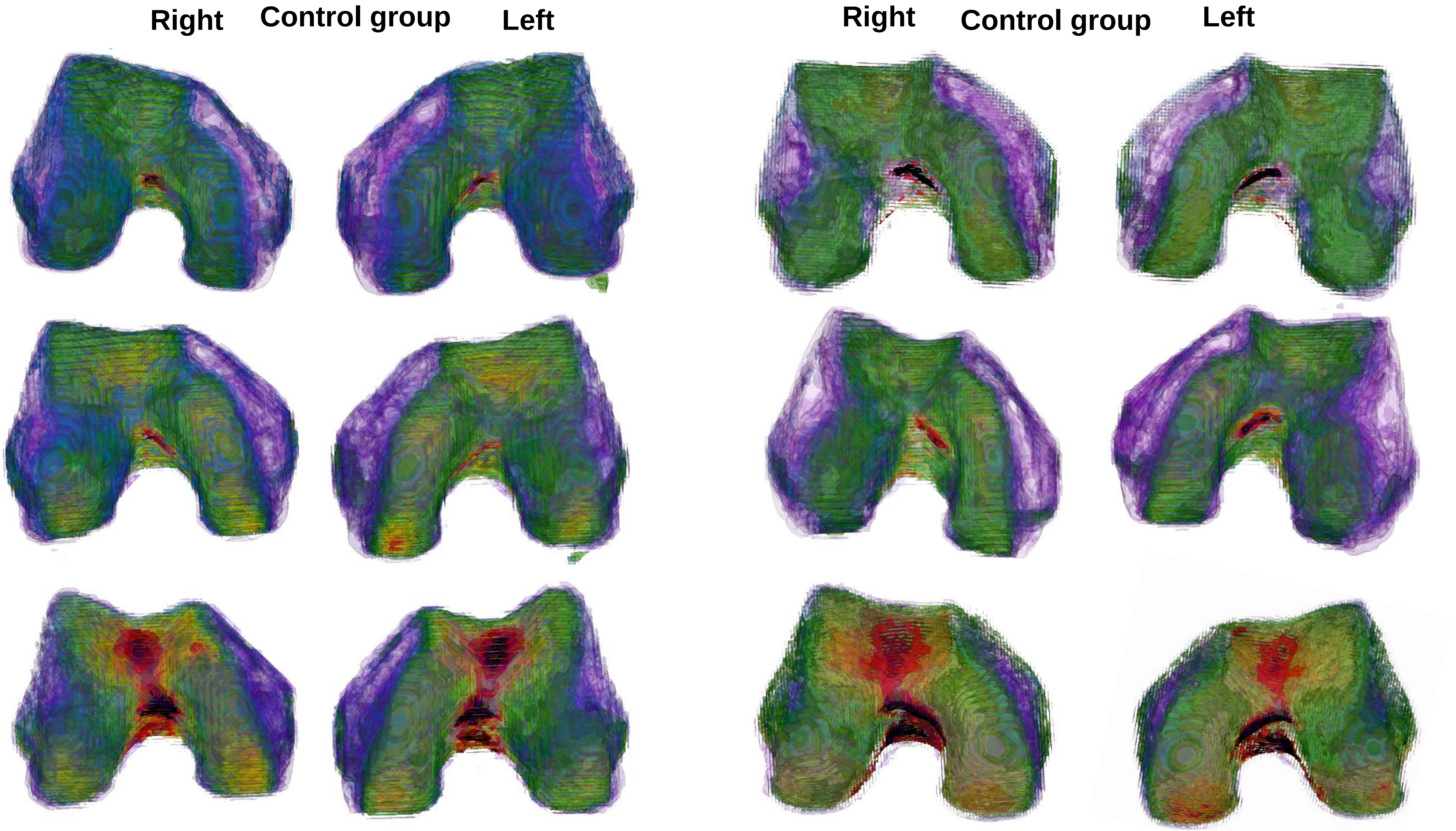
Right
Control group
Left
Right
Control group
Left

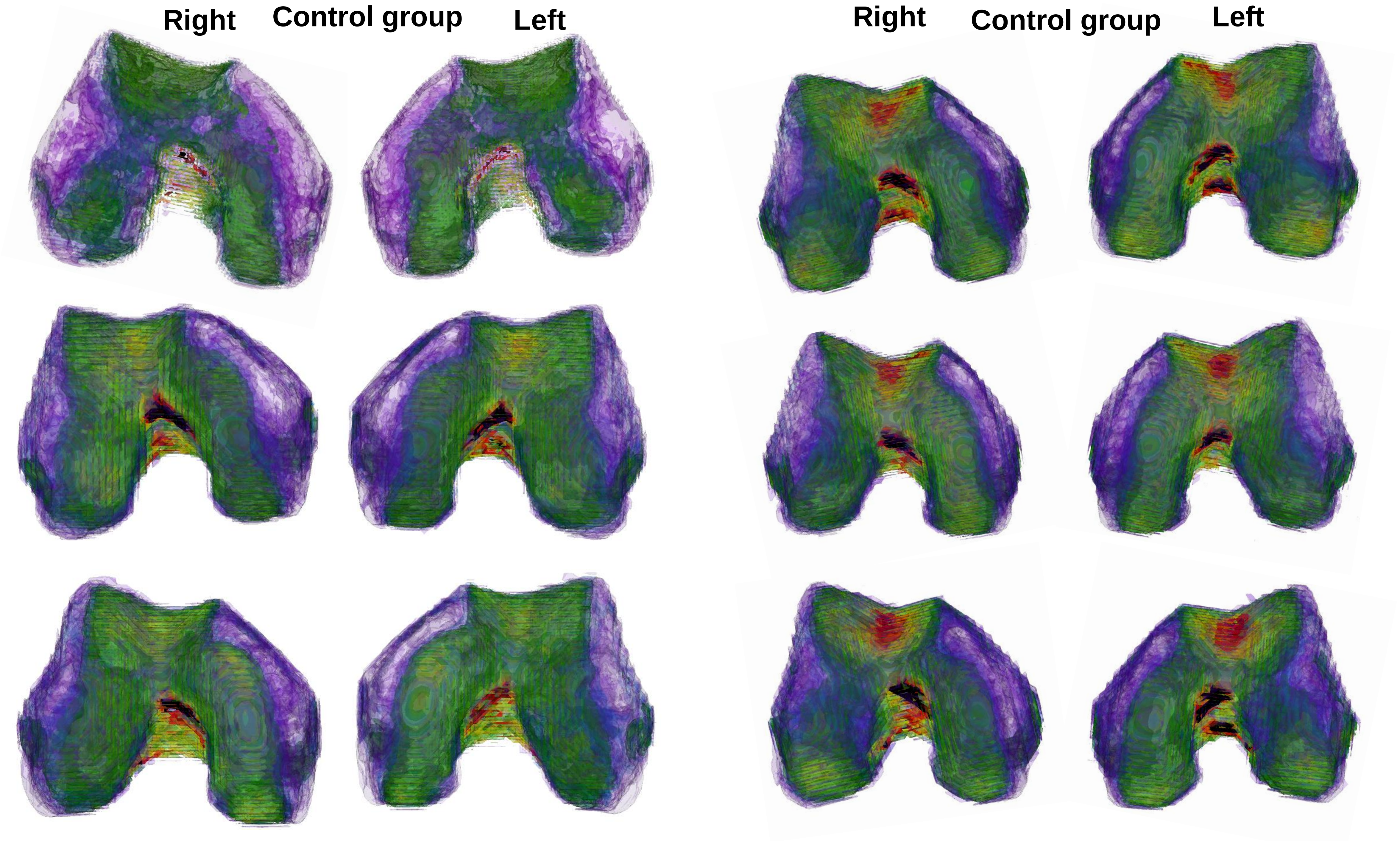
Right
Control group
Left
Right
Control group
Left

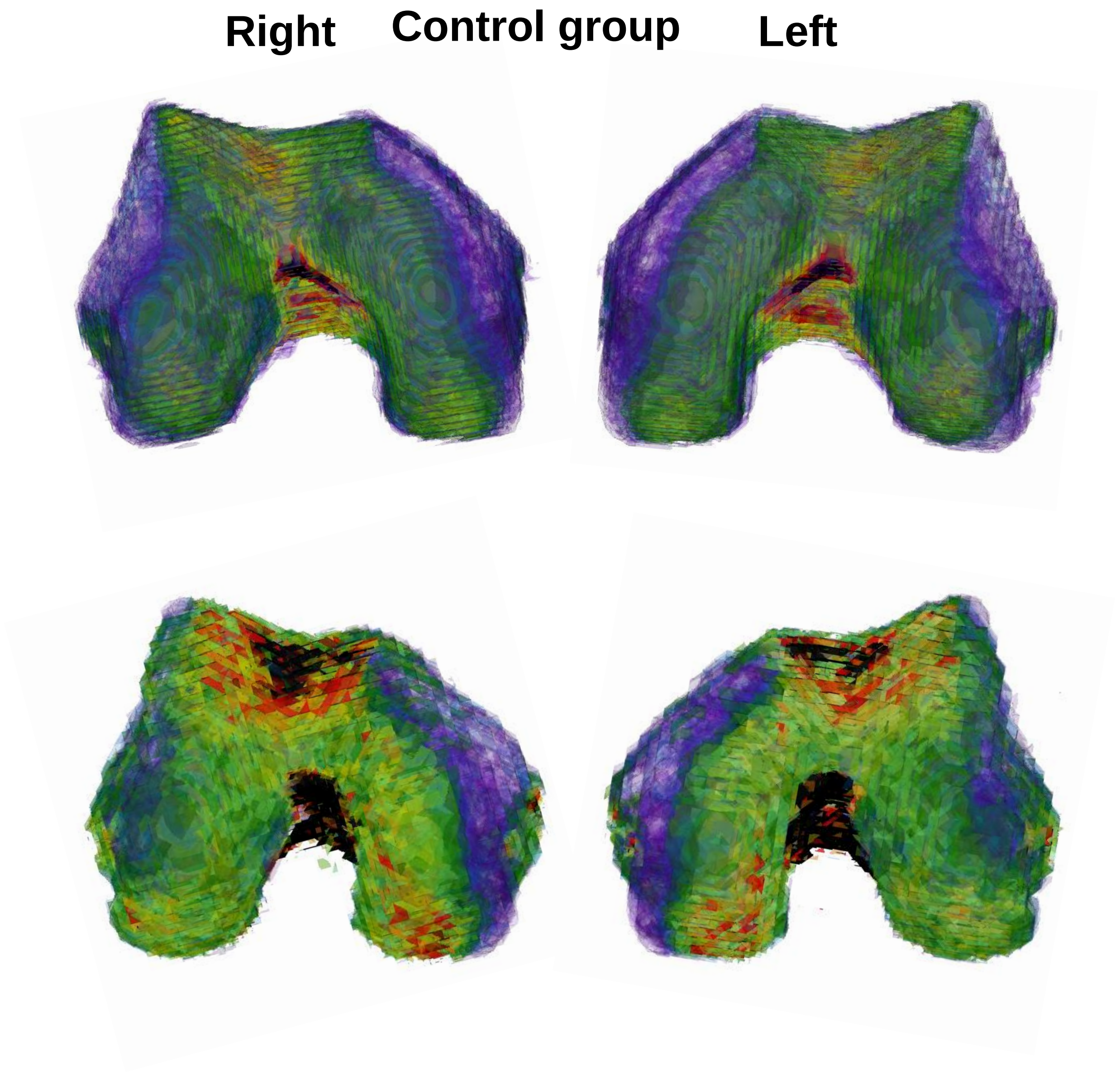
Right
Control group
Left

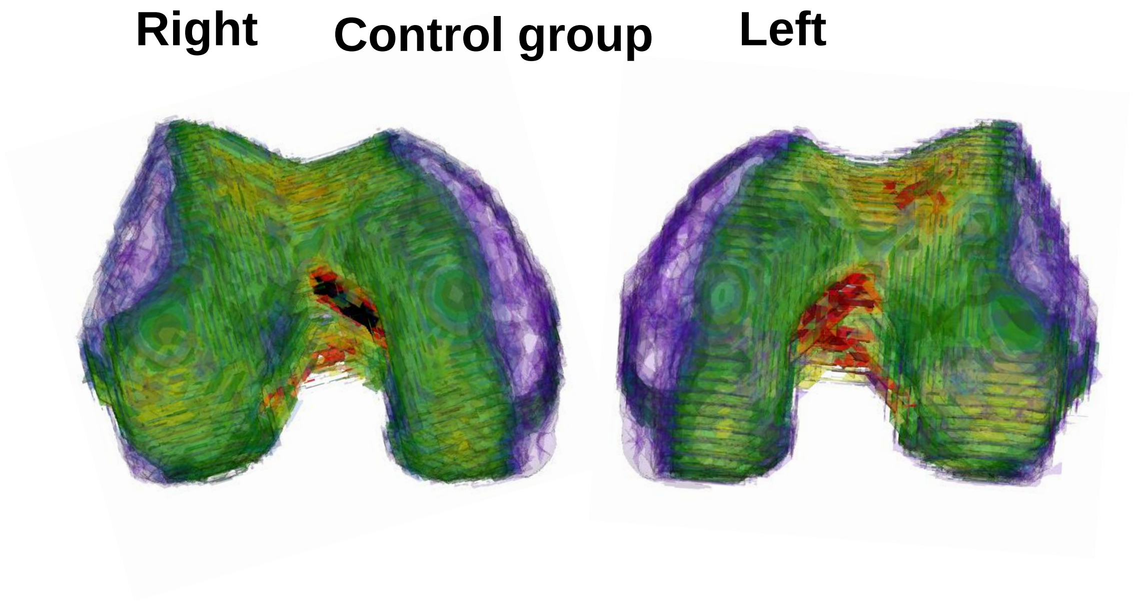
Right
Control group
Left

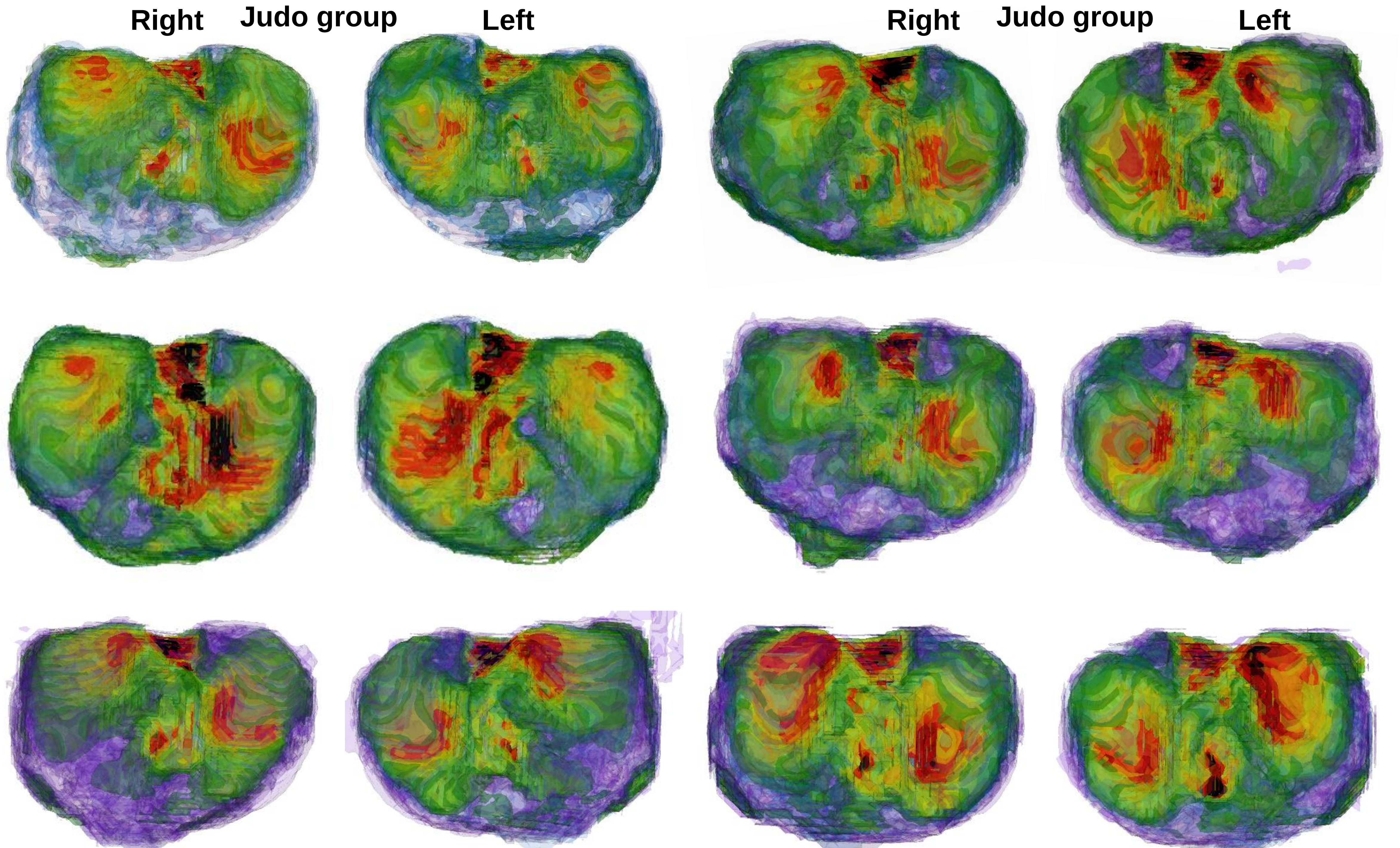
Right
Judo group
Left
Right
Judo group
Left

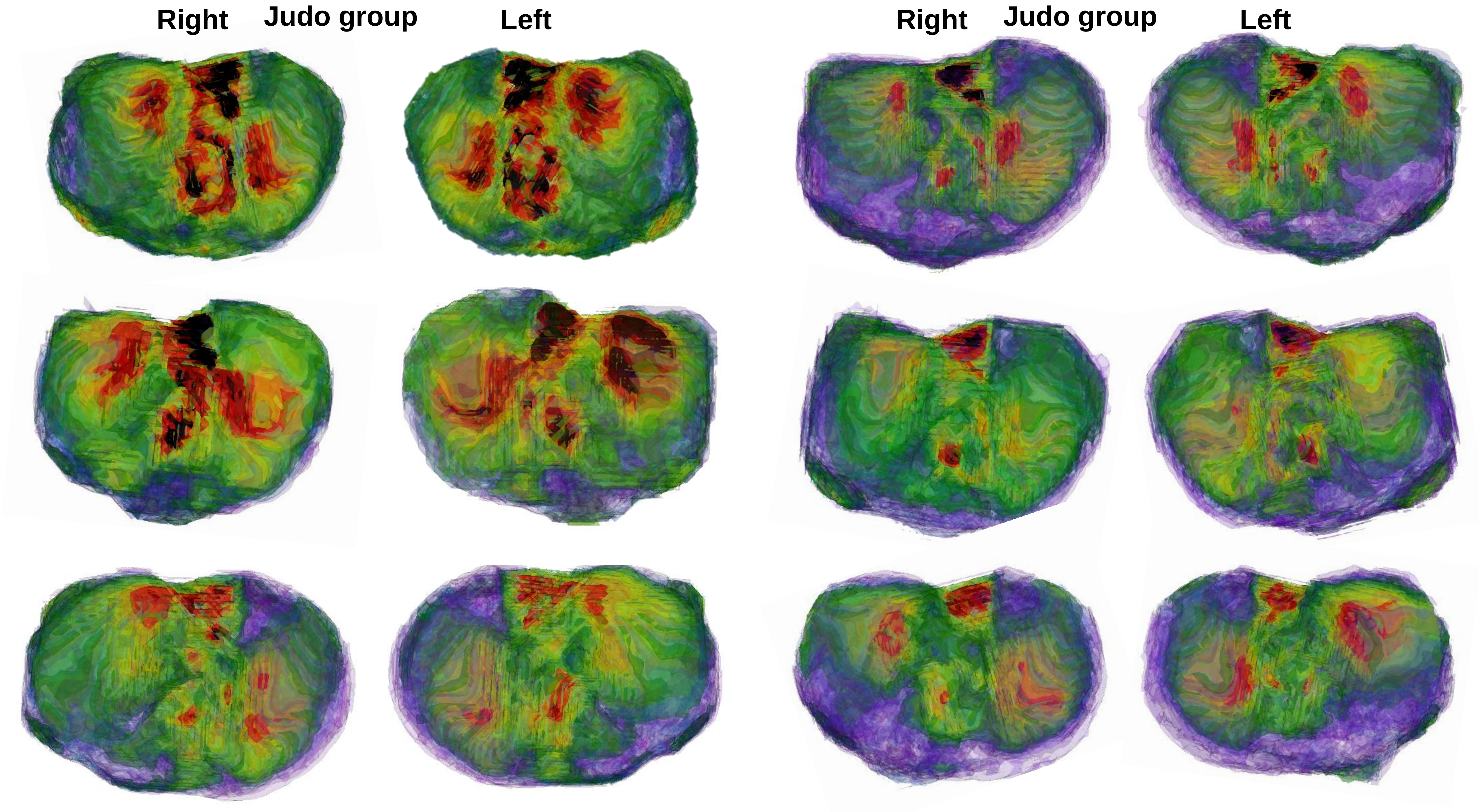
Right
Judo group
Left
Right
Judo group
Left

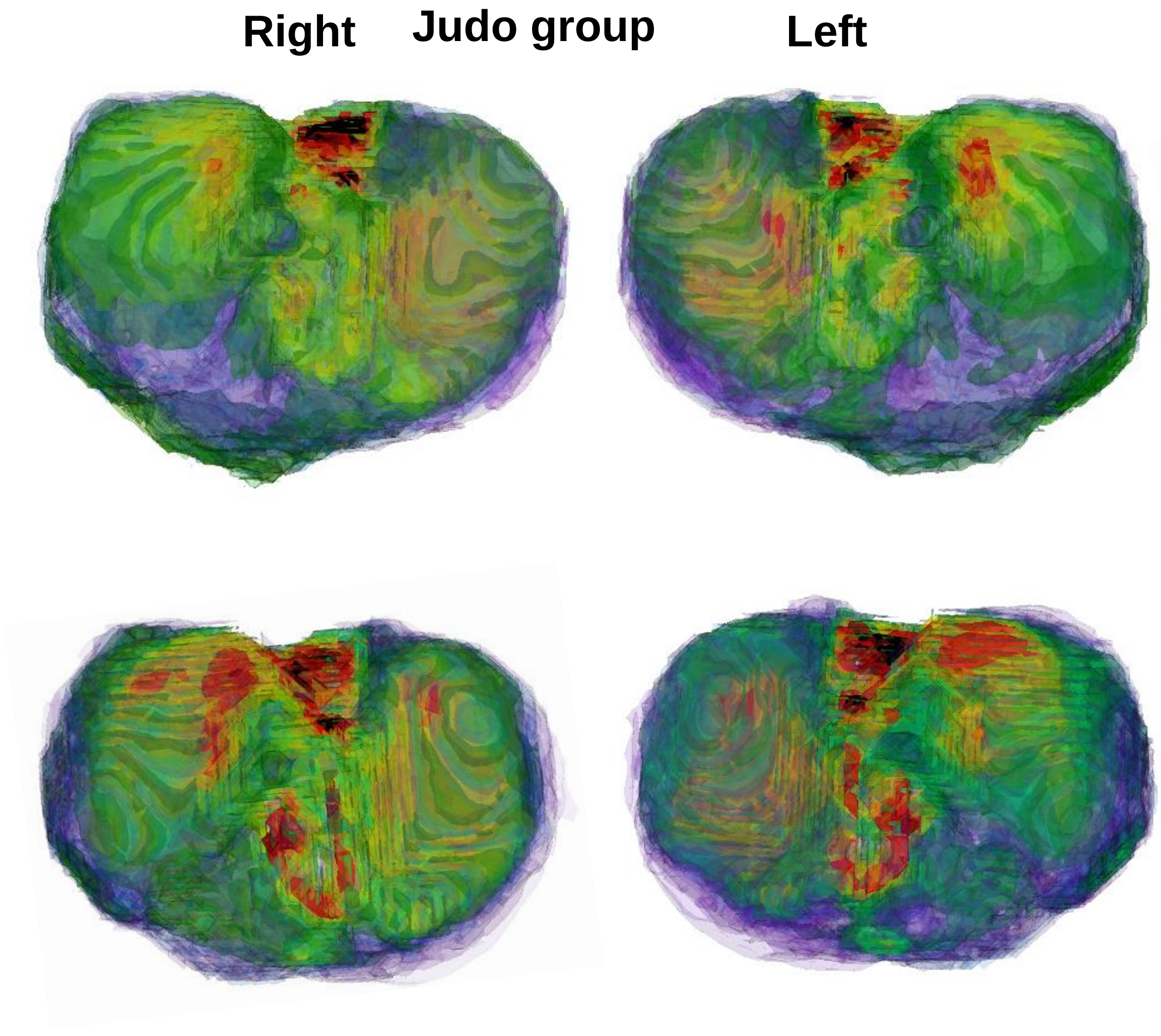
Right
Judo group
Left

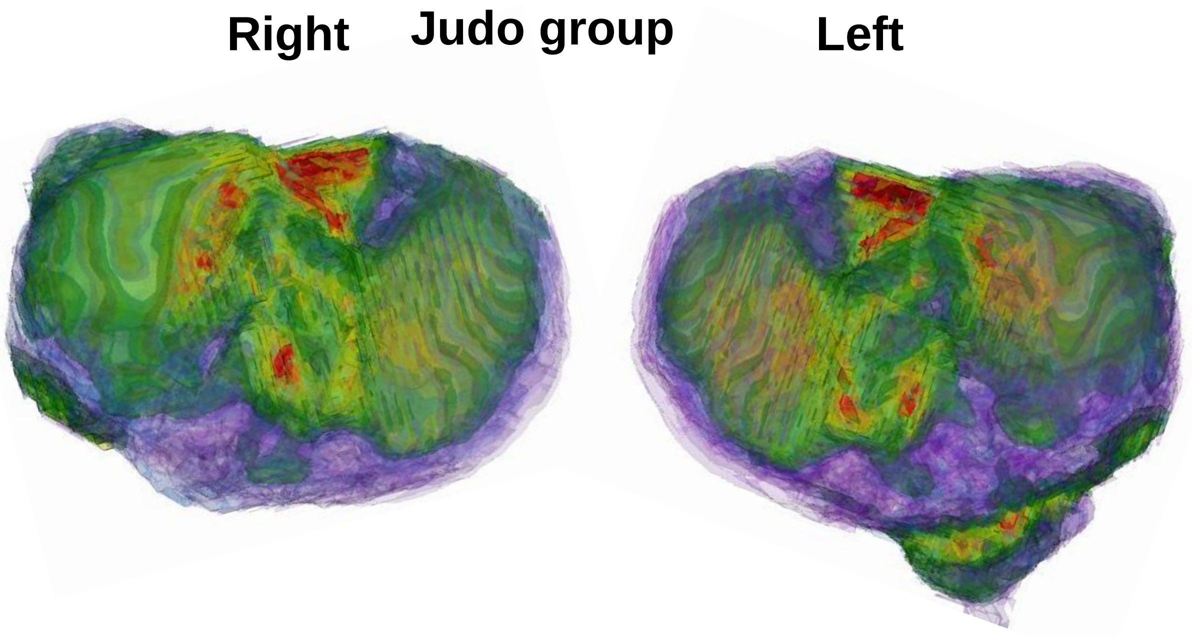
Right
Judo group
Left

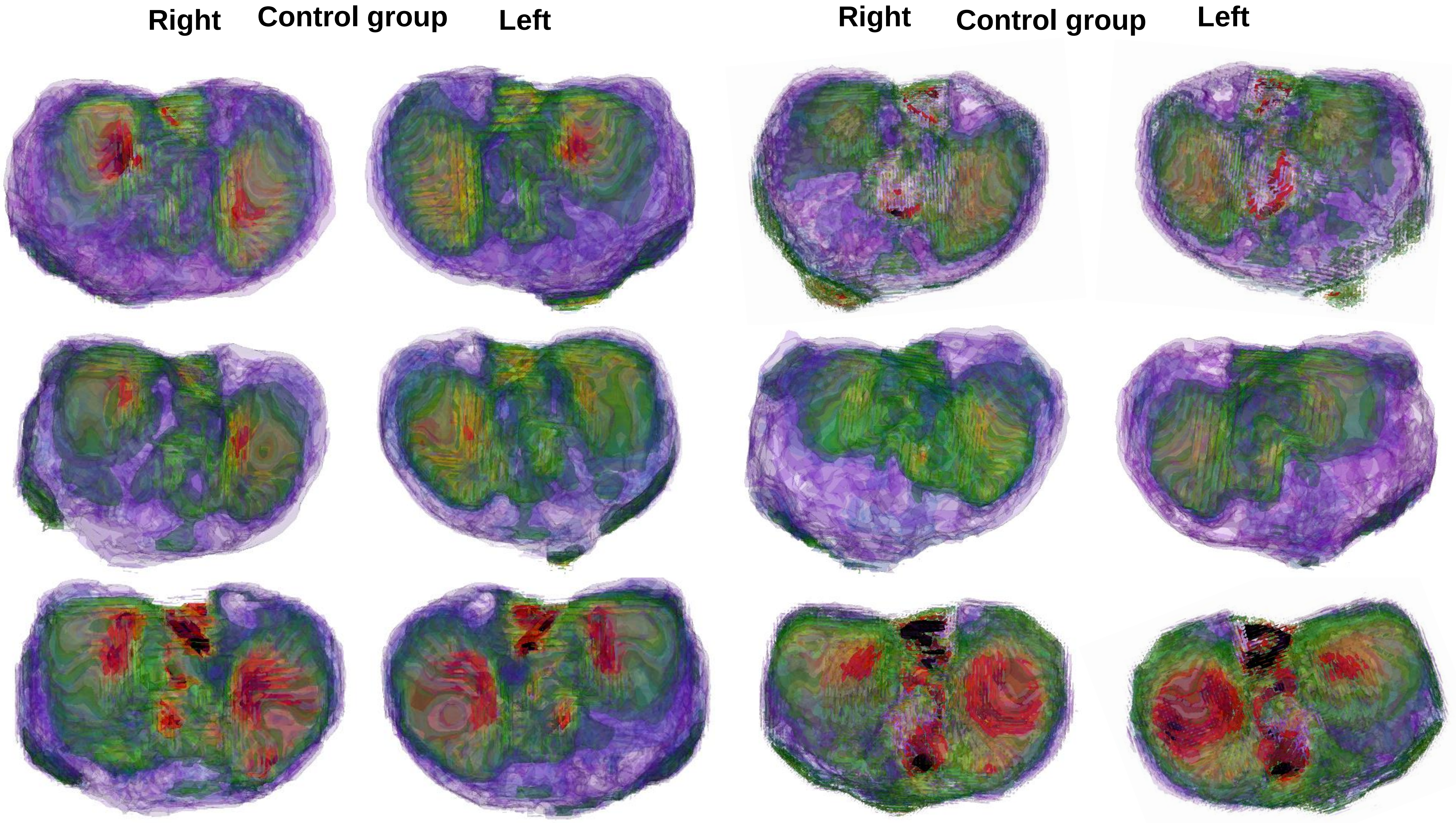
Right
Control group
Left
Right
Control group
Left

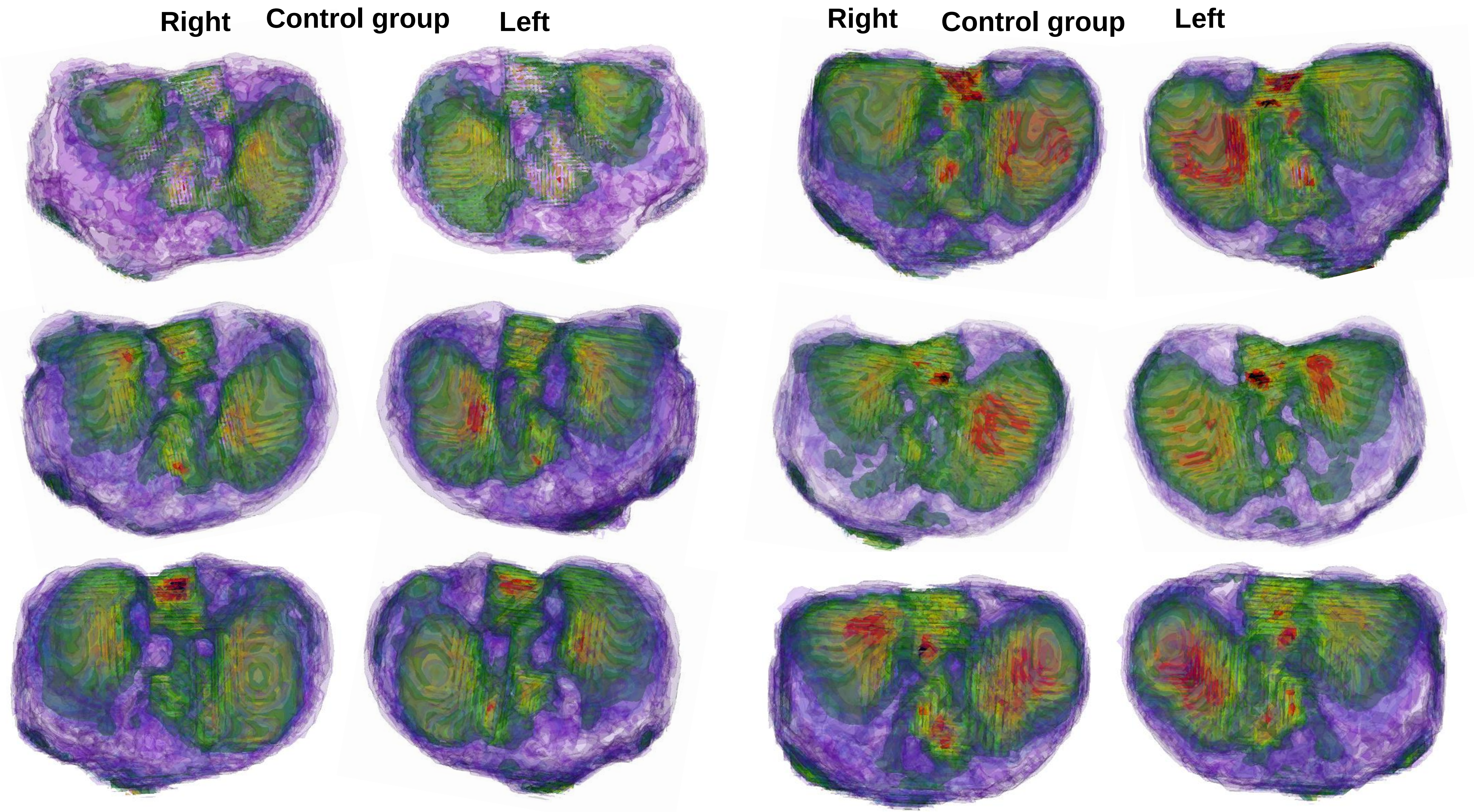
Right
Control group
Left
Right
Control group
Left

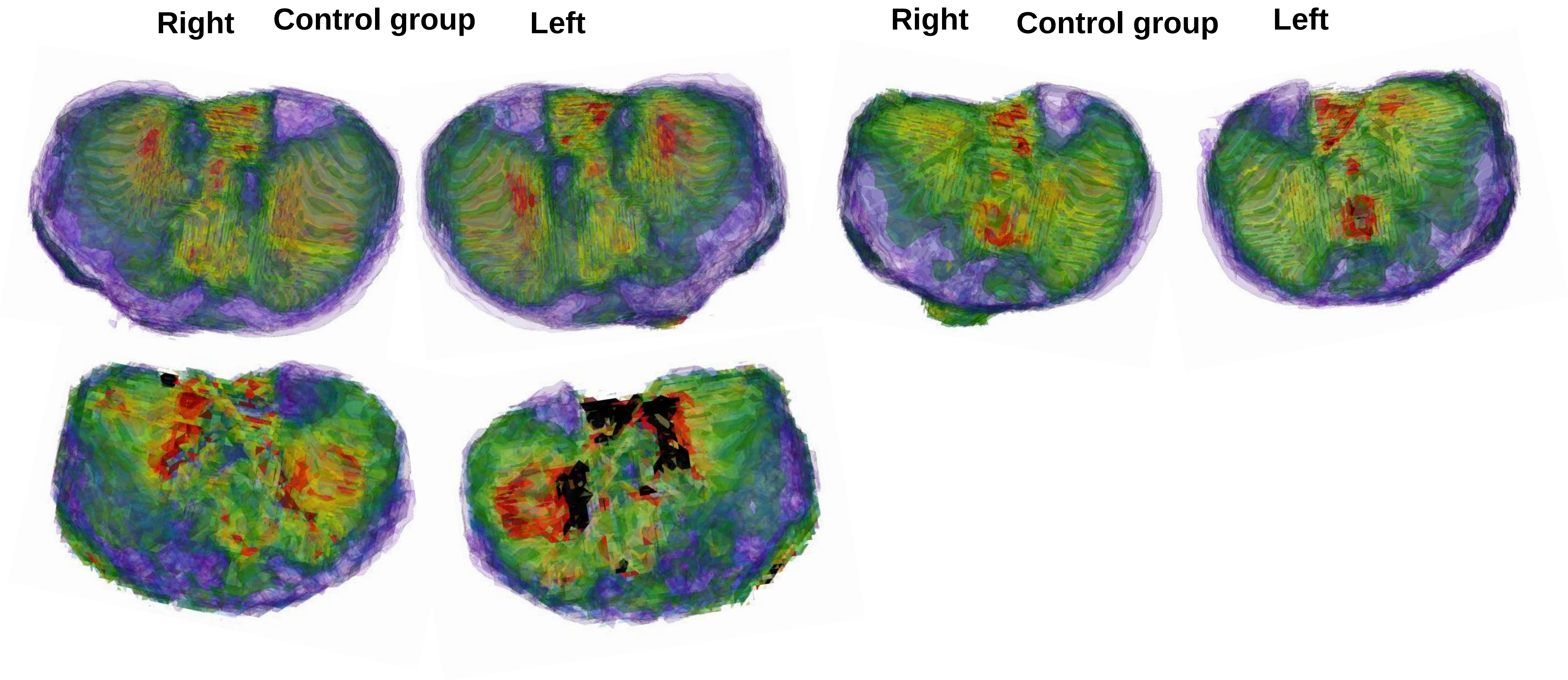
Right
Control group
Left
Right
Control group
Left
