# Supplementary figures and images for: Variation characteristics of stress distribution in the subchondral bone of the knee joint of judo athletes with long-term stress changes
Source: Front Endocrinol (Lausanne). 2023 Jan 24;13:1082799. doi: 10.3389/fendo.2022.1082799 (PMC9909959; doi:10.3389/fendo.2022.1082799)

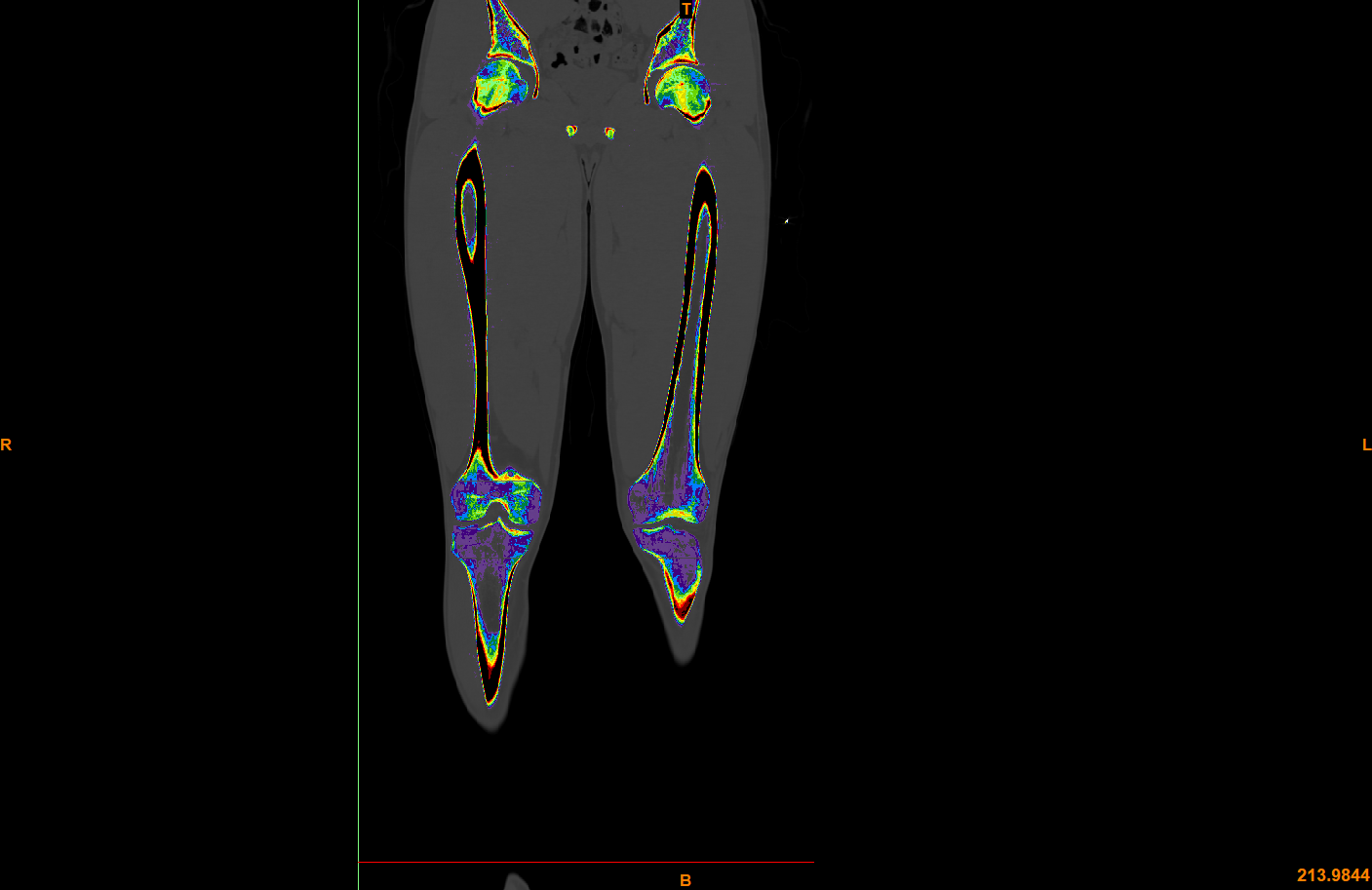

Supplement: Supplementary file 1 [file DataSheet_1.zip › supplement material-figure 1 CT image of total low limbs in CTOAM.png]
